# A systematic review of reported reassortant viral lineages of influenza A

**DOI:** 10.1186/s12879-015-1298-9

**Published:** 2016-01-05

**Authors:** Amy Pinsent, Christophe Fraser, Neil M. Ferguson, Steven Riley

**Affiliations:** Department of Infectious Disease Epidemiology, MRC Centre for Outbreak Analyses and Modelling, School of Public Health, Imperial College London, London, UK

**Keywords:** Influenza, Reassortment, Reassortant, Evolution, Genomic data, Reporting, Pandemic

## Abstract

**Background:**

Most previous evolutionary studies of influenza A have focussed on genetic drift, or reassortment of specific gene segments, hosts or subtypes. We conducted a systematic literature review to identify reported claimed reassortant influenza A lineages with genomic data available in GenBank, to obtain 646 unique first-report isolates out of a possible 20,781 open-access genomes.

**Results:**

After adjusting for correlations, only: swine as host, China, Europe, Japan and years between 1997 and 2002; remained as significant risk factors for the reporting of reassortant viral lineages. For swine H1, more reassortants were observed in the North American H1 clade compared with the Eurasian avian-like H1N1 clade. Conversely, for avian H5 isolates, a higher number of reported reassortants were observed in the European H5N2/H3N2 clade compared with the H5N2 North American clade.

**Conclusions:**

Despite unavoidable biases (publication, database choice and upload propensity) these results synthesize a large majority of the current literature on novel reported influenza A reassortants and are a potentially useful prerequisite to inform further algorithmic studies.

**Electronic supplementary material:**

The online version of this article (doi:10.1186/s12879-015-1298-9) contains supplementary material, which is available to authorized users.

## Background

Although the importance of reassortment as a mechanism for driving the emergence of novel influenza genotypes (with pandemic potential) has been recognized for many years [1–3], an understanding of this process at the population level has been impossible until recently because of a lack of genomic data. However, the increasingly widespread availability of whole-genome sequencing [4] has permitted a rapid expansion (Additional file 1: Figure S1) in the number of high quality descriptive evolutionary studies that rely on genomic data. Reassortants with important public or animal health consequences can now be confirmed within weeks and their evolutionary history explained soon afterwards [3, 5, 6]. Rapid progress is also being made in the laboratory to determine the specific pathways to increased virulence and transmissibility, caused by the exchange of whole gene segments that occur as a result of reassortment between different influenza A subtypes and across host types [7–9].

Pathogen-dynamic studies of reassortment have previously focused on specific influenza subtypes [10–12], hosts [4, 13, 14] or evolutionary events [3, 15, 16]. More recently, rates of reassortment within a particular viral lineage have been estimated [17, 18] and high-risk areas in which reassortment may occur have been explored [19]. However, broader descriptions of patterns of reported reassortment remain lacking in the literature. Indeed, while it is hypothesised that there are likely to be biases in the reporting of reassortants from different hosts and geographical regions, to our knowledge there is no statistical or quantitative evidence in the literature to suggest a strong bias towards certain hosts over others. An increase in the number of publicly available genomes of influenza A across all hosts and subtypes has motivated an expanding literature on the algorithmic detection and classification of reassortant viruses [20–27], through phylogenetic analysis or via statistical models of genetic distance. However, these methods can be very computationally intensive and are consequently only tested on small subsets of available data. Therefore, before additional methodological advances can be made, a logical first step is to collate and understand the patterns of reported peer-reviewed claims of reassortment. If improved algorithms with greater accuracy are to be developed, it is important to understand the patterns of reassortment that have been reported and published to date, in order to be able to assess the findings from computational studies of reassortment. Indeed the dataset collated in this study could be viewed as a training set from which existing and new algorithmic methods could be refined, developed and validated.

Throughout this review we define a reassortant viral lineage of influenza as follows: “a circulating virus or group of viruses in which at least one gene segment of the influenza isolate is located in a discordant position on a phylogenetic tree relative to its other gene segments”.

We identify the first reported isolate (FRI) of each reassorted lineage by: a) systematically reviewing the literature and b) using the publicly available sequence data associated with the reported isolates to decide between conflicting apparent first-reports.

Having identified a set of unique FRIs, we examined the extent to which geographical [1] and host [28, 29] drivers of pandemic emergence may or may not be reflected in the frequency with which novel reassortants have been reported in the literature, within one publicly available sequence database. We subsequently compared the phylogenetic relationships between gene segments in the reassortant set and Same Size Random Subsets (SSRS) of a control drawn randomly from all isolates for which whole genome data was available in GenBank. In this analysis we focus on two specific host-subtype combinations that are currently considered as primary threats to human health, avian H5 and swine H1.

We do not suggest that the reassortants reported in the literature represent an unbiased sample of reassortants that occur in nature. However, this set of isolates for which a claim has been made and for which evidence has been provided do represent the extent of our current knowledge on the complete reporting of reassortants.

## Methods

### Search strategy and selection criteria

We performed the analysis and search according to the recommendations of the Preferred Reporting Items for Systematic Reviews and Meta-Analyses (PRISMA) statement [30], which is available in Additional file 2. A protocol for the review is provided in Additional file 3.

Electronic searches were conducted in PubMed (MEDLINE) and Web of Knowledge (all databases) to identify relevant articles. Articles published before 1st of July 2013 using the terms “influenza and (reassortment or reassortant)”, were included in the review. No restrictions with respect to language, publication period or study design were applied. We also searched the Cochrane Library and OpenGrey database, however, so few articles were returned, we chose not to consider them as we were primarily interested in peer reviewed claims of reassortants.

Studies included were those which reported the identification of a naturally occurring, unique novel influenza A reassortant viral lineage, which was not already in the database. No restriction was applied with respect to different influenza A subtypes. The article presented clear phylogenetic evidence to indicate that at least two gene segments had been sequenced, with GenBank accession numbers for gene segments provided. Full details are shown in (Additional file 1: Figure S2).

Studies excluded were those that provided GISAID [31] accession numbers, however checks were made to see if the isolates had also been uploaded to GenBank. Studies for which phylogenetic evidence was only given for one gene segment were excluded (to ensure that unobserved reassortment events had not occurred). Studies were excluded if the report identified an interspecies transmission event or a co-infection event that did not result in reassortment, along with laboratory studies of reassortment. Articles in which reassortment was proposed but did not explicitly identify the isolates in the text or supporting information were excluded, because without isolate information we could not assess patterns of reassortment by host, geographic region and year. Articles in which reassortant isolates were identified as part of a methodological project were excluded (however these were often only conducted with a small number of isolates). Also excluded were articles which reported a ressortant that had been reported elsewhere, for example many articles were based on a secondary analysis of a previously reported strain such as 2009 pandemic H1N1. A flow chart indicating the number of papers removed at each stage is indicated in (Additional file 1: Figure S2).

All titles and abstracts were examined; if the title was not sufficient for a clear rejection then the full article was obtained for all those which suggested the identification of a first reported isolate that was a reassorted influenza A virus.

Despite many papers making reference to reassortment, far fewer papers reported unique novel reassortant lineages accompanied with clear genomic data. Up to 1st July 2013, we identified 3,754 articles in multiple searches. Of the 3,754 articles, we excluded 3,101 articles on the basis of manually searching the abstract and title because it was clear that there was no claim of a new naturally occurring reassortant, leaving 653 articles claiming to have identified a novel reassortant of influenza A. The main text of all articles were then reviewed and we included only those that: provided phylogenetic evidence of a natural influenza A reassortment event; with explicit isolate name; and the suggestion that two or more gene segments had been uploaded to GenBank; leaving a total of 209 articles for data extraction (Additional file 1: Figure S2).

### Data extraction

The data extracted from each article was as follows: first author, year of publication, title of article, year of reassortment event (year associated with isolate), host type, the geographic region the isolate was sampled from (provided in the strain name), complete strain name, and whether the emergence of the isolate was due to an inter or intra-subtype reassortment event. If the latter piece of information was not explicitly stated in the text we assessed the phylogenies presented in the paper to identify whether isolates clustering with the reported FRI were the same subtype and whether this was consistent across all eight genes. Accession numbers for all isolates were identified and recorded along with the number of gene segments presented in the study. We subsequently restricted the analysis to isolates for which sequence data for all eight gene segments was available. This was done to ensure we could discount the suggestion that unobserved reassortment had occurred on gene segments not sequenced. All isolates are listed in Additonal file 4: Database S1. Alignments were made for each gene segment for all isolates using ClustalX2 [32].

### Quality assessment

We used the same criteria to classify reassortant viruses across all papers ensuring consistency. By subsequently restricting the analysis to whole genomes we reduced the chance that reassortment may have occurred on other gene segments but not observed by the authors. Quality of the sequences themselves was not assessed, nor the robustness of the trees on which the reassortant report was made, as we were interested in capturing as many reports as possible. The analysis did not aim to verify or falsify the claim of a reassortment event that was reported, only to summarise the information available in the existing literature.

### Removal of highly homologous isolates

**Fig. 1:**
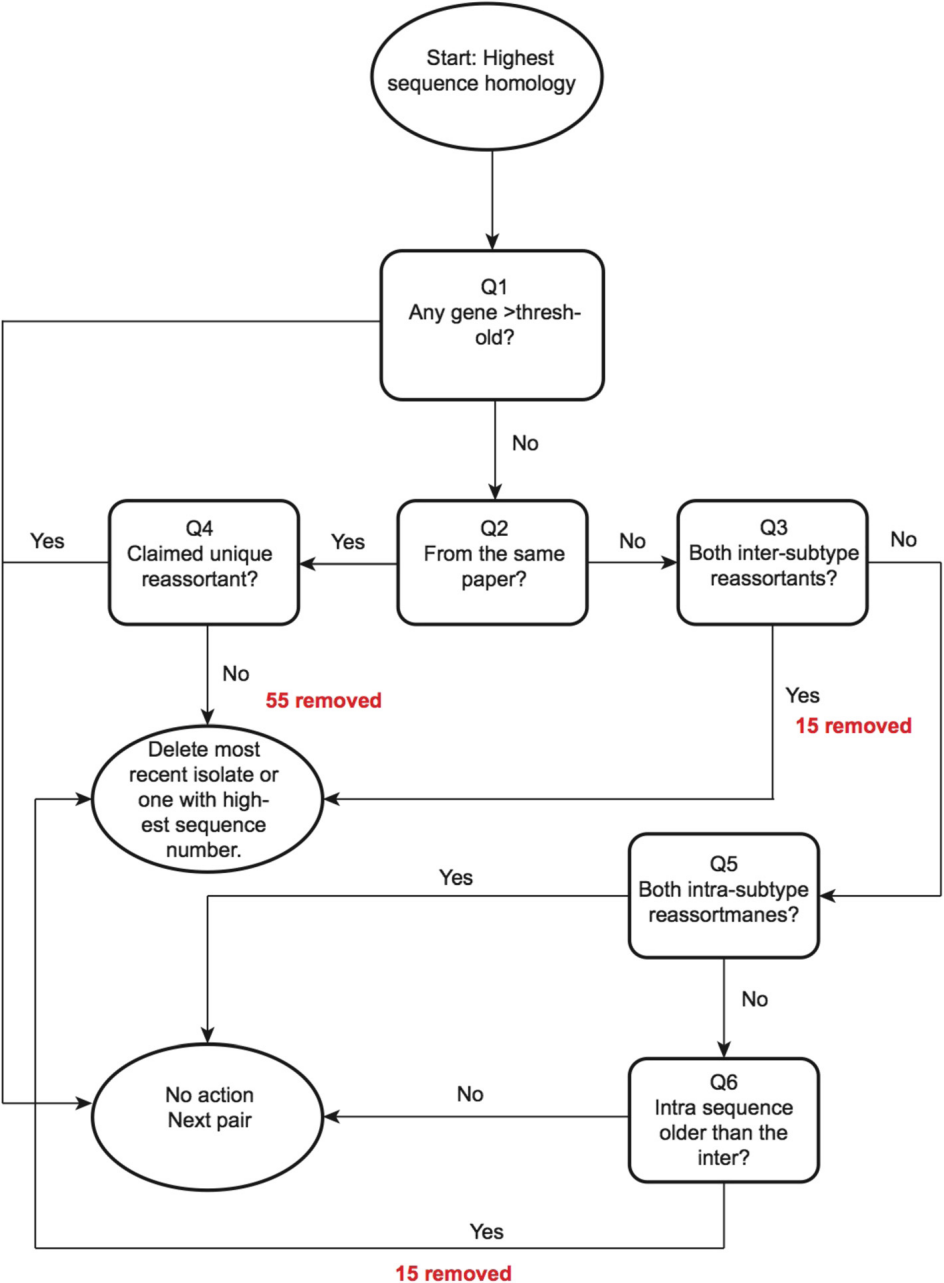
Decision tree applied to remove suspected duplicate strains from the unique set of FRIs identified in the literature. Data was first ranked according to whole genome sequence homology, from lowest to highest. The pairs were evaluated individually, starting with the most genetically similar through the following criteria indicated in the figure (detail provided in Methods). Numbers in red at deletion decision points indicate the number of isolates that were removed at that stage

A pairwise comparison of each reassortant virus genome against all others in the dataset was computed using R software [33]. The number of base pairs available for comparison and the number that differed between two sequences were computed, together with the percentage identity between all gene segments within each strain pair, hence we computed the Hamming distance between each strain pair. An additional set of exclusion criteria were then developed to remove highly homologous isolates from the dataset. These may have been missed in the initial screening of the papers, or they may be very similar isolates reported in different papers. We did not attempt to verify or falsify claims from peer reviewed literature, only assess whether highly homologous isolates had been included. A pairwise comparison of each strain’s genome against all others in the dataset was computed, to remove highly homologous isolates. Starting with the most similar pair based on sequence homology, we applied a decision tree (Fig. 1) to the first ~1500 most similar pairs, to decide whether a strain should be deleted from our set (Fig. 1). The number of isolates removed at each stage is indicated in red on Fig. 1 and is recorded clearly in Additional file 4: Database S1.

We firstly assessed whether any differences in genetic Hamming distance between gene segments were greater than the selected threshold value. This was 40 base pair (bp) differences on a non HA/NA gene for 2 reported inter-subtype reassortants isolated between 0–3 years of each other. For isolates greater than 3 years apart, this threshold was increased to 80 base pairs on a single non HA/NA gene. For comparisons with intra-subtype reassortants, at least 30 bp variation on a non HA/NA gene needed to be observed, this threshold was increased to 60 bp on a non HA/NA gene if isolates were greater than three years apart.

The thresholds were decided based on the result that on average 40 bp variation across the genome for an influenza isolate is the average amount of diversity expected in a given season [34]. We therefore assumed that most of the variation would accrue on HA or NA during this time, and not the internal genes. As such, we felt that greater than 40 bp on a non HA or NA gene over a 3 year period would be sufficient to determine dissimilarity between isolates.

We assumed more variation would be expected in isolates that had undergone inter-subtype reassortment in comparison to intra-subtype reassortment; hence the threshold for intra-subtype reassortants was lowered to greater than 30 bp variation. The thresholds were then doubled when 3–6 years between isolates was observed. If any comparison showed greater than this threshold variation on a gene segment both strains of the pair were kept in the set. If not, we moved to question 2 and progressed through the decision tree (Fig. 1). If we chose to delete a strain, all pairs that contained that strain were deleted from the list of pairs. Numbers in red at deletion decision points indicate the number of isolates at that stage for that reason (Fig. 1). With the exception of 1 isolate (A/SW/SK/12-71/2009) no deletions were made on the basis of genetic distance on HA or NA. Sensitivity of the results to the deletion of these isolates is presented in Additional file 5.

### Collection of comparator data

Comparator data for the regression analysis was gathered from GenBank on 5th July 2013. We collected all meta and sequence data for all available isolates, for all regions, years (up to and including 2013) and host-types, provided that there was at least some sequence information for all eight influenza A gene segments. This represented 20,781 unique isolates. This provided a dataset from which all reassortants may have been reported and allowed us to describe the odds of an isolate being reported as an FRI. We assumed that all other isolates did not represent FRI genotypes. The same data was used for the same size random subset (SSRS) comparisons, for Swine H1 and Avian H5.

### General additive logistic model

The final FRI data (646 isolates) and the comparator data were used to develop a general additive logistic model which was implemented using R software [33] with the mgcv package [35]. We calculated the odds ratio (OR) for each of the covariates (host, region and year) being reported as a reassortant. Hence we use reported reassortant isolates as the response variable and the three covariates (host, region and year) as the predictor variables. The effect of covariates were considered significant when the p-value was < 0.05 (and the 95 % CI of the OR did not overlap one (no effect)). The quality of each general additive logistic model was assessed using the Akaike Information Criterion (AIC) score.

### Genetic analysis

We used all data in GenBank for which sequence data was available for all eight segments of all influenza A isolates across all host types (the same denominator data used for the regression analysis). We chose non-FRI isolates randomly from this data to compare the genetic diversity (calculated as the Hamming distance between isolates) observed in the reassorted data to random samples from GenBank. Ten same size random samples (SSRS) were taken from the data for H1 swine and H5 avian isolates (that were non-FRIs) to generate comparative distributions of genetic diversity (based on Hamming distance), using data from the HA gene segment only. 113 HA1 gene segments were randomly sampled from 1,110 swine H1 whole genomes, and 118 HA5 gene segments were randomly sampled from 2,298 avian H5 whole genomes, this sampling process was repeated ten times. One of each of the ten SSRS’s was used to generate a comparative phylogeny with the reassorted data for both HA1 Swine and HA5 avian gene segments. Maximum likelihood phylogenetic trees were generated using FastTree [36] assuming a generalized time reversible model of nucleotide substitution.

### Assessment of bias

We conducted additional stratified regression analysis to examine the robustness of the results obtained from the statistical analysis. The analysis was a meta-analysis of reported reassortants. We adapted traditional funnel-plots [37], designed to assess publication bias, and created similar funnel-like plots. However, we did not synthesise independent estimates of the odds ratios from each of the studies included in the analysis, this was not possible as many studies only reported a single isolate as reassortant, not the number of isolates they sequenced in their study in order to identify the reassortants reported. As such it was not possible to synthesise independent estimates for each study. Nor did we re-analyse the reassortants that had been previously been reported to determine whether the reassortant was genuine. In our funnel-like plots, instead of analysing the effect size of each individual study, we analysed the effect size for each year of data that we had. As such, the year in which the sequences were isolated were equivalent to separate studies in a traditional funnel-plot analysis. This plot is presented as in Additional file 1: Figure S5.

### Sensitivity analysis

We performed a sensitivity analysis to attempt to include articles that did not provide and make available explicit isolate names for the reassortants that they claimed. The maximum information was obtained from the 13 papers that were excluded (Additional file 1: Figure S2). From these papers, an additional 125 possible FRIs were inferred, the majority of which came from aquatic birds in the USA. To ensure that excluding these isolates did not impact the main findings of the analysis, we re-performed the regression analysis (describe in the results section below) to include the extra 125 FRIs. The results are presented in Additional file 6.

## Results

### Whole genome restriction and suspected duplicate removal

The 209 articles identified in the literature search made reference to 876 isolates that were available in GenBank with some sequence data available for at least two gene segments. We excluded 145 isolates because sequence data were not available for all eight gene segments, leaving a total of 731 claimed unique reassortant isolates with sequence data in GenBank (Additonal file 3: Database S1).

We suspected that duplicate reports of the same reassortant genotypes may have been included in the dataset generated, quite possibly from the same paper. A database of all 266,815 unique combinations of pairs of the 731 claimed unique reassortant lineages was generated. For each gene segment of each isolate pair we computed the degree of sequence homology between the isolate pair, before sorting the pairs by overall sequence homology (see Methods for full details). A decision tree was applied, starting with the most similar isolate pairs, to determine whether one or other of the pair was a duplicate isolate and should be deleted (Fig. 1). We identified 85 duplicates (55 of which were initially reported as having the same genotype as one another in the same research article). This process resulted in a final set of 646 unique FRIs (Additonal file 3: Database S1).

### Probability of reassortment by host, region and year

Crude ratios of the number of FRIs to the number of available full-genome sequences suggested patterns in host and geographic region of isolation (Table 1). The highest proportion of first reported reassorted viruses were isolated in swine with poultry also generating a high numbers of reassortant viral lineages relative to the total number of sequences available for each host type in GenBank. Compared to isolates from non-human hosts, very few reported reassortants were identified in the published literature for humans, despite the large number of human isolates that have been sequenced (Table 1). For geographical regions, China and Europe reported the highest number of FRIs, while Russia, the Middle East and South America reported very few. However, there was also a much lower total number of isolates available in GenBank for these regions.

**Table 1:** Number of first reported reassortant isolates stratified by host and region

	Africa	Asia	Australia	China	Europe	Japan	Middle East	Russia	South America	US	Total (Host)
Aquatic Bird	1 (38) *(0–5.2)*	25 (674) *(16.2–36.5)*	1 (100) *(0–5.22)*	113 (953) *(94.0–134.1)*	22 (294) *(13.8–33.1)*	6 (75) *(2.24–12.4)*	0 (6) *(0–2.7)*	3 (51) *(0.6–8.2)*	0 (11) *(0–3.1)*	67 (4,289) *(52–84.9)*	238 (6,491) *(209.1–269.0)*
Equine	0 (2) *(0–1.68)*	0 (2) *(0–1.6)*	0 *(0–0)*	0 (13) *(0–3.2)*	1 (20) *(0–4.97)*	0 (6) *(0–2.7)*	0 (1) *(0–0.9)*	0 *(0–0)*	0 (5) *(0–2.6)*	0 (54) *(0–3.5)*	1 (103) *(0–5.4)*
Human	0 (63) *(0–3.5)*	4 (1,426) *(1.1–10.2)*	4 (1,016) *(1.1–10.2)*	10 (915) *(4.8–18.3)*	11 (1,020) *(5.5–19.5)*	5 (168) *(1.6–11.4)*	0 (40) *(0–3.5)*	0 (110) *(0–3.6)*	0 (495) *(0–3.6)*	28 (4,382) *(18.6–40.4)*	62 (9,635) *(47.5–79.4)*
Other Avian^a^	1 (19) *(0–3.3)*	5 (82) *(1.6–11.2)*	0 (3) *(0–2.1)*	38 (387) *(27.2–51.1)*	0 (17) *(0–3.31)*	0 (31) *(0–3.4)*	1 (25) *(0.2–6.5)*	0 (4) *(0–2.4)*	0 (1) *(0–0.9)*	0 (95) *(0–3.6)*	45 (664) *(33.1–59.5)*
Other^b^	0 (0) *(0–0)*	1 (53) *(0–5.33)*	(0) *(0–0)*	6 (90) *(2.2–12.5)*	0 (5) *(0–2.6)*	0 (0) *(0–0)*	0 (0) *(0–0)*	0 (1) *(0–0.9)*	(0) *(0–0)*	2 (221) *(0.2–7.1)*	9 (370) *(4.1–16.9)*
Poultry	6 (177) *(2.2–12.8)*	28 (448) *(18.7–39.9)*	0 (9) *(0–3)*	70 (622) *(55.2–87.1)*	4 (121) *(1.1–9.9)*	0 (35) *(0–3.5)*	2 (90) *(0.2–7)*	2 (18) *(0.2–6.2)*	0 (4) *(0–2.4)*	8 (239) *(3.4–15.5)*	120 (1,763) *(100.0–142.5)*
Swine	0 (2) *(0–1.6)*	21 (166) *(14.2–31.6)*	0 (3) *(0–2.1)*	56 (508) *(43.7–72.6)*	18 (166) *(10.1–26.3)*	5 (30) *(1.69–10.4)*	(0) *(0–0)*	(0) *(0–0)*	2 (9) *(0.2–5.4)*	69 (868) *(55–87.4)*	171 (1,752) *(147. 3–197.0)*
Total (Region)	8 (301) *(2.8–14.2)*	84 (2,851) *(68.0–104.7)*	5 (1,131) *(1.6–11.6)*	293 (3,488) *(262.5–327.8)*	56 (1,643) *(41.6–71.2)*	16 (345) *(9.2–25.6)*	3 (162) *(1.1–10)*	5 (184) *(1.6–11.4)*	2 (525) *(0.2–7.1)*	174 (10,148) *(150.2–202.6)*	646 (20,778) *(600.0–699.0)*

All regions and hosts for which data on reported reassortants was identified. Data indicated in the top row of each box shows the number of reassortants identified, to the left in brackets is the total number of isolates for each region and host type available in GenBank for all eight gene segments. Data below in italics indicates the 95 % binomial confidence intervals. Other avian^a^ as a host group includes isolates from pheasants, sparrows, quails, partridges, chukkars. Other^b^ as a host group includes reassortants from environmental samples- surface water and canines

**Fig. 2:**
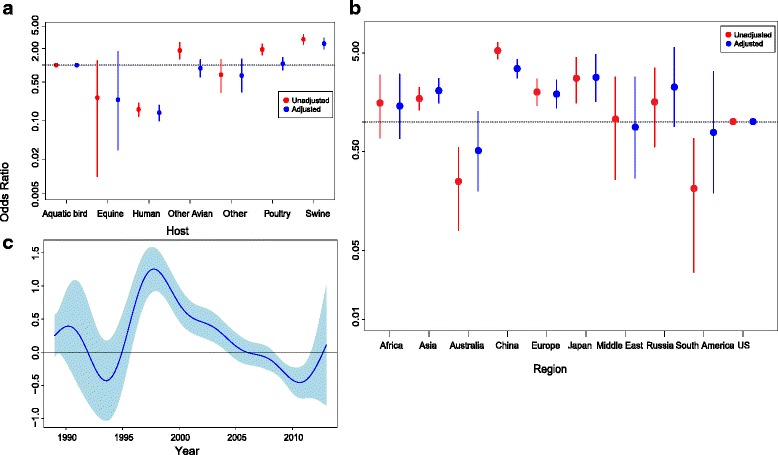
Crude (*red*) and adjusted (*blue*) odds ratios for the rate of reporting inter and intra-subtype reassortants by region (**a**), host (**b**) and year (**c**). The dashed line at 1.00 on **a** and **b** represents the baseline level of risk, vertical bars crossing the dashed line indicate no effect at the 5 % level. The vertical lines above and below each point indicate 95 % confidence intervals. Adjusted odds are from the general additive logistic model of region and host as factors and year of isolated as a smooth term (**c**) [35]. Blue areas of **c** show 95 % confidence interval for smooth term for the contribution to the odds from the year of isolation. The univariate analysis where year was treated as a categorical variable is presented in Additional file 1: Table S1. Blue areas of **c** show 95 % confidence interval for smooth term for year of isolation

To account for correlations between these three covariates, we constructed a set of multivariate general additive models to assess the probability that a given publically available genome was reported as an FRI. The model that included all three covariates (host, region and year of isolation) received that greatest statistical support, as assessed by the Akaike Information Criterion (AIC) (Fig. 2). When assessing inter and intra-subtype reassortants together, China, Europe and Japan remained as significant predictors for reporting reassorted viruses in the final model, with the odds of reporting reassortment events isolated from China higher than Europe and the baseline, OR: 3.45 (95 % CI: 2.78–4.28), OR: 1.92 (95 % CI: 1.38–2.65), OR: 2.80 (95 % CI: 1.62–4.84) for China, Europe and Japan respectively (Fig. 2b blue points & Additional file 1: Table S1). A reduced risk of reporting reassortants from Australia was observed, but was not retained in the final model (Additional file 1: Table S1). The significance of reporting reassorted viruses from poultry or other avian hosts observed in the univariate analysis was not retained in the final model with OR: 1.07 (95 % CI: 0.83–1.37) and OR: 0.89 (95 % CI: 0.63–1.25) respectively. The significance of reporting reassortant viruses in swine hosts was retained in the final model OR: 2.48 (95 % CI: 1.99–3.09) (p-value <0.001).

A significant increase in the odds of reporting reassorted viruses from different years of isolation was identified in the categorical univariate analysis, with year as a categorical variable (1997–2002, Additional file 1: Table S1). Given the trend observed in the univariate analysis (Additional file 1: Table S1), we fitted a smoothing spline to year in the general additive model, which was statistically significant (*p*-value <0.001, Fig. 2c).

We also developed 2 separate multivariate general additive models for isolates reported as inter or intra-subtype reassortants so that they could be analysed separately (Additional file 1: Figures S3 and S4) to assess whether the odds of reporting inter and intra-subtype reassortants varied across the covariates assessed.

### Reporting inter-subtype reassortants

For the univaraite analysis of inter-subtype reassortants, other avian and swine showed an elevated risk of being hosts in which inter-subtype reassortants would be reported: OR: 1.84 (95 % CI: 1.25–2.65) (*p*-value < 0.01) and OR: 2.81 (95 % CI: 2.23–3.53) (*p*-value < 0.001), respectively. However, only swine as a predictor was retained in the final model (Additional file 1: Figure S3a and Table S2). We observed in the univariate and multivariate analysis that the odds of a reassortant isolate being reported from China was higher than the baseline and Europe (Additional file 1: Figure S3b and Table S2). Years 1997–2003 highlighted a significant increase in the odds of reporting reassorted viruses in the univariate analysis (with year as a categorical variable), a smoothing spline was fitted to year in the multivariate analysis (*p*-value < 0.001) (Additional file 1: Figure S3c).

### Reporting intra-subtype reassortants

Other avian, poultry and swine had an elevated odds of being identified and reported as intra-subtype reassortant isolates, however only poultry and swine were retained in the final model OR 2.28 (95 % CI: 1.51–3.44), (*p*-value <0.001) and OR: 2.64 (95 % CI: 1.70–4.08), (*p*-value <0.001) respectively (Additional file 1: Figure S4a and Table S3). China, Africa, Asia and Japan were significantly more likely to report reassortant isolates, relative to the baseline (USA). However, only China and Japan were retained in the final model. China had the highest odds of reporting and publishing isolates (Additional file 1: Figure S4b and Table S3). Only the year 1997 was an indicator of excess risk of reporting reassortants in the univariate analysis (with year as a categorical variable). Years 2007–2011 were indicators of reduced risk. A smoothing spline was fitted to year as a co-variate in the multivariate model which was statistically significant (p-value <0.001) (Additional file 1: Figure S4c).

### Assessing for bias

We conducted additional stratified analysis by year and made funnel-like plots (Methods). There did not appear to be a systematic correlation between the standard error of our estimate for the log odds of a swine virus being reported as reassortant (Additional file 1: Figure S5). However, particularly in China there did appear to be a trend such that in years (or groups of years) in which sequence data for fewer isolates was uploaded to GenBank, the odds of an isolate being reported as reassortant were higher (Additional file 1: Figure S5b). One of the groups of years for which this effect was apparent was the most recent period study, 2011–2013 (group 3, Additional file 1: Figure S5b). It seems probable that reassortant isolates are more likely to be uploaded and reported quickly relative to non-reassorted isolates. This was also seen for Band 1 in China, again, it is likely that when resources were limited and sequencing was expensive, only the isolates of most interest were likely to be sequenced and uploaded to GenBank.

### Relative diversity of the reassorted set for illustrative gene segments

#### Swine HA, H1

**Fig. 3:**
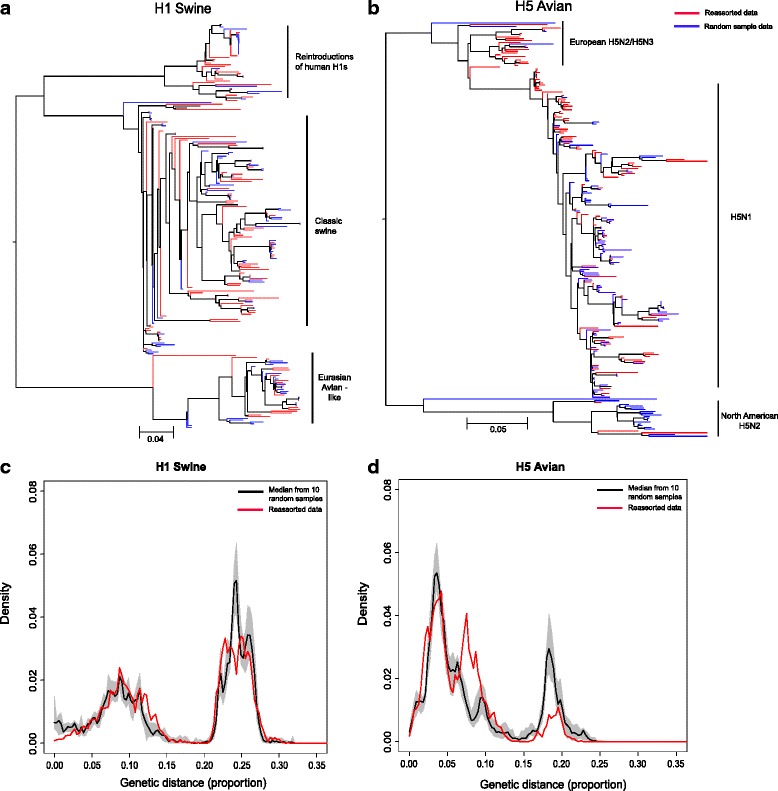
Maximum likelihood phylogenies and distributions of pairwise genetic similarity shown as a density of Hamming distance for H1 Swine, H5 Avian. Maximum likelihood phylogenies illustrating the phylogenetic relationship between 1 same size random sample and the FRI data for **a** H1 Swine and **b** H5 Avian. The type of data on each tree is indicated by the tip colour, red tips show FRI data and blue tips show data from 1 same sized random subset. Clustering of particular clades is indicated on the tree. Trees were mid-point rooted. Distribution of pairwise genetic similarity shown as a density of hamming distance for **c** H1 Swine, **d** H5 Avian. Isolate comparisons for each host-subtype combination from the reassorted set are indicated in red, and the median across ten same size random subsets of isolates from GenBank for each host/subtype pair is indicated in black. The grey shaded area represents the 10^th^ and 90^th^ percentile across the ten same size random subsets. Labelled phylogenies are provided in Additional File 7

The reporting of reassortment appeared to be more frequent in the clades of H1s recently re-introduced from humans than in the avian-like clade (Fig. 3a). The robustness of this observation was tested by computing the distribution of diversity for ten randomly chosen same sized random subset (SSRS) of 113 publicly available whole genomes of swine H1 isolates and comparing it with the distribution of diversity for swine H1 FRIs (Fig. 3c).

The overall distribution of genetic diversity for swine H1 was characterised by two distinct peaks. The first peak at lower diversity was made up of many pair-wise comparisons within the large and diverse Classic swine H1 clade, as well as comparisons within the Eurasian avian-like clade itself and comparisons within the clade of re-introduced human H1s (Fig. 3a). Little difference between the random subsets and reassorted data was observed as shown by the overlap of the two datasets on the histogram (Fig. 3c). The second peak reflected comparisons between Classic swine and the two other main groups: the Eurasian avian isolates and the group made up of recently reintroduced human H1 isolates (Fig. 3c). The difference between the red line and the grey area at the second peak in Fig. 3a suggested that the observation of increased reporting of reassortment in the clade of recently reintroduced human H1 was not an artefact of the selection of the SSRS (Fig. 3a and c).

#### Avian HA, H5

We conducted a similar analysis as above for Avian H5 and found increased reporting of reassortment in European H5N2/H5N3 compared to North American H5N2 (Fig. 3b and d). We note that the second reassortant isolate in this cluster was an isolate from Taiwan. However, all other isolates in this clade were from North American. The distribution of genetic diversity was characterised by three distinct peaks. The first peak at a lower Hamming distance reflected comparisons within the diverse avian H5N1 subtype; here there was a clear overlap between the reassorted data and random samples, suggesting a high degree of genetic similarity. Phylogenetic comparisons of the reassorted and random sample data (Fig. 3b) highlighted that a large clade of European H5N2 and H5N3 isolates had generated a high number of reassortants, whilst this was not true for H5N2 isolates collected in North America. The European H5N2 /H5N3 reassortants generated the second peak of high frequency comparisons in the distribution of diversity (when these isolates were compared to H5N1 isolates) (Fig. 3d, red line). However, a very low frequency of comparisons between the random sample datasets were observed at this point suggesting that a high number of European H5N2/H5N3 that are sequenced and uploaded to GenBank are also reported as reassortant. The absence of overlap in genetic similarity at the second peak between the random subsets and reassorted data provides evidence to support this claim. The third peak at ~ 0.2 dissimilarity reflected a high frequency of random sample comparisons of North American H5N2 isolates, however only a very small number of FRI comparisons were observed (Fig. 3b and d). This suggested that while North American H5N2 isolates appear to be frequently uploaded to GenBank they are not frequently reported as reassortant isolates.

## Discussion

In this analysis we have identified 646 distinct claims of first reported reassortant isolates for influenza that have been published in the peer reviewed literature, for which some sequence data were available for all eight gene segments. Of these, 416 were reported as inter-subtype reassortants and 230 were intra-subtype reassortants (Additonal file 3: Database S1). Together, these represent just 3 % of the total 20,781 full genomes available in GenBank (up to July 2013). Elevated odds of isolates from certain host types being reported as reassortant were no longer significant, with the exception of swine hosts, once region, host and year were accounted for using a general additive logistic model. This result was supported by additional sensitivity analysis that was conducted using articles previously excluded because they did not explicitly identify claimed reassortants in the text (Additional file 6).

To our knowledge, we have provided the first quantitative estimates on the odds of reporting reassorted viruses using data from the peer reviewed published literature. Despite the risks of publication bias, our findings in all three general additive models provide substantial quantitative support to suggest swine are hosts in which reassortment frequently occurs [28, 38, 39]. We have shown statistically significant support for China as a region in which the odds of reporting reassorted viruses were above the baseline region (USA), where the odds of reassortment for China were higher than Europe, supporting China as a potential hot-spot of influenza diversity [39, 40]. These results appeared to be robust to a high increase in the number of FRIs added to the USA and to aquatic birds (Additional file 6).

Reported inter and intra-subtype swine H1 and avian H5 reassortants were analysed together in the genetic analysis. However, despite differences in the regression analysis between the two types we suggest that little difference in this analysis would be apparent if the data were stratified. 40 of the 113 swine H1 isolates were reported as intra-subtype reassortants most of which were H1N1 isolates, the removal of these isolates would therefore only reduce the number of comparisons made at low levels of genetic diversity. We suggest that this would also be true for avian H5 isolates, 49 of the 118 avian H5 isolates were reported as intra-subtype reassortants. However, all isolates were H5N1; therefore the removal of H5N1 isolates would only reduce the frequency of comparisons at low levels of genetic diversity. In general, genetic distributions of same-sized random subsets of all available complete genomes for each host and subtype pair showed high concordance; however disparities in the distributions of diversity for avian H5 highlighted an under-representation of subtypes which have generated a high number of ressortants; suggesting that the tracking and surveillance of avian H5N2 and H5N3 is of paramount importance for future surveillance. The collective analysis of this data provides a valuable step in helping to develop targeted surveillance strategies to identify “hot-spot” hosts and regions for reassortment.

Although it could be argued that there is no value in describing the pattern of reported reassortants, we disagree. Sample bias is an issue for most studies of viral evolution. Almost every conclusion about the location and timing of ancestral populations is subject to unquantifiable uncertainty because of known gaps in surveillance and uncertainties about the sampling process where surveillance is present. In order to begin the process of addressing these gaps, we suggest it is important to describe patterns in what has been reported, and then assess the robustness of specific conclusions against known issues in sampling, as we have tried very carefully to do here.

An alternative approach to the systematic review presented here would be to develop a robust algorithm (with high sensitivity and specificity) and to apply it to all publicly available genomes. Ideally, we would compare the output of such an analysis with the systematic review, to understand both detection and publication biases. However, to our knowledge, existing phylogenetic algorithms appear to have low sensitivity (since the number detected is much lower than found by our systematic review) [20, 24, 26, 27], and the most robust algorithms would be difficult to apply to all available data, since they rely on assumptions of analysing random representative samples and are computationally expensive to apply to very large datasets [21].

de Silva et al. [26] identified and reported 52 reassortants that they were confident about. Of these, they report that 12 had previously been reported in the literature. However, when comparing this to the results from our systematic review we identified 17 reassortants that were also identified by the algorithm in our study, while another 5 isolates identified as reassortant by the algorithm were included in the sensitivity analysis presented in Additional file 6. de Silva et al. [26] analysed 1,670 full genomes which they believed to be representative of the 9,284 sequences, this is just under half of the number of sequences considered in our review. This suggests that in comparison to the high number of reassortants identified in the review, the algorithm applied had low sensitivity. However, evidenced by the high number of reassortants identified by the algorithm that have not been reported in the literature information is lacking on both sides. This is why we suggest performing the systematic review presented here is an important first step in helping to develop more informative algorithms. It is interesting to note that the large majority of reassortants missed by the review were reported from avian hosts, this supports the suggestion that due to the difficulty in analysing and interpreting reassortment events that have occurred in avian hosts, they are reported less frequently. Equally, while no obvious differences in the region of isolation were identified between the reassortants identified in this review and those identified with the algorithmic approach. It could be suggested that if China as a region was more likely to report and upload reassortants to GenBank in comparison to other regions, this may lead to a bias, resulting in the inflated odds of reporting reassortants from China observed in this study. Furthermore, it is also important to consider that de Silva et al. [26] only used 1,670 representative sequences. Therefore it is possible that reassortants detected by the algorithm may have the same representative genotype as those identified and reported in the review presented here, but are represented by isolates with different names.

At this stage, though subject to publication and detection biases, we think that the systematic review presented here (and detailed in Additional file 4: Dataset S1) collates one of the most comprehensive collections of identified reassortants to date. A full comprehensive phylogenetic analysis of all publically available sequence data would represent the best possible knowledge available on the occurrence of reassortment in nature, however at this point in time no such analyses is available. To our knowledge, we have not seen a full phylogenetic analysis of all GenBank data that includes all host types, regions and years of isolation. Therefore highlighting patterns of reported reassortment we feel is still a valid analysis and is extremely important. Also, we suggest that a systematic review of claimed reassortants would be a valuable resource with which to calibrate any automated reassortment detection algorithm.

We did not include claims of reassortants for which genomic data were only uploaded to the GISAID database [31], which is a clear limitation to our study. However, we note that only 9 isolates were not included in the FRI set because they had only been reported in GISAID and not GenBank, and that there were only 2,430 full genomes in GISAID available at the time the study was conducted, in comparison to the 20,781 full genomes available in GenBank.

It cannot always be assumed that the year, host or region an FRI was detected actually reflects the host, place or time that the reassortment event occurred.

It may be possible to overcome the bias of using region of isolation as a proxy for region in which the reassortment event occurred by undertaking phylogeographic analysis, such that the location of the most recent common ancestor (MRCA) of each reassortant isolate could be identified. Equally, we have used year of isolation of each reassortant lineage to suggest the time at which the reassortment event occurred. To improve upon this it may be possible to use detailed phylogenetic dating methods to obtain an estimate of the tMRCA for each reassortant viral lineage and then compare this to the date of isolation.

A bias inherent in the analysis was due to the selection criteria at the initial stages of the review, whereby we specified that reassortant isolates must be identified explicitly in the article or supporting information. This may have created a bias against large-scale studies used to investigate the frequency of reassortment, particularly in aquatic birds. Wille et al. [41] reported a high frequency of reassortment in Anas platyrhychos but did not explicitly identify the isolates in the text. Additionally, Dugan et al. [42] do report a high frequency of reassortant isolates, but do not explicitly identify all reassortant isolates identified. Furthermore, the heavy mixing in gene phylogenies when analysing samples from aquatic birds suggests highly complex reassortment events. This data can be technically challenging to interpret and it can be difficult to precisely define the reassortment events that have occurred. This could result in fewer publications associated with these particular reassortment events due to the challenges associated with interpreting the results, resulting in a secondary bias relating to the publication of reassortant isolates collected from aquatic birds. Additionally, bias towards reporting infection from livestock in which disease is very apparent, could lead frequent reporting of these infections. However, in natural systems such as wild birds disease is rarely apparent this may lead to a reduced sampling and reporting of infection from these hosts. However, the results presented in this analysis appear robust to a large increase in the number of aquatic bird isolates reported as reassortant (Additional file 6). This may also lead to biases in the reporting of reassortants from different geographic regions if different regions have a higher propensity to sample more routinely or only when livestock present with symptoms. While this is a limitation, we feel that the analysis performed here could not be done if the isolates names were not explicitly reported. We have shown that all hosts except swine whose reassortants are reported in the literature have a lower risk than aquatic birds of having their reassortant isolates reported. It is possible that there may be a systematic bias in the uploading of sequences to GenBank, where more routine uploading of samples from aquatic birds occurs. While isolates from swine hosts may only be uploaded when they are perceived to be interesting. As such, a higher portion of all swine isolates uploaded to GenBank may be classified as FRIs, which may not be true for aquatic bird samples.

## Conclusion

Sampling of influenza in both humans and animals is highly heterogeneous in time, space and host-type. Therefore, we do not assert that patterns identified in this analysis, such as an increased odds of reporting reassortants in swine or China, reflect the overall global transmission network for influenza. However, the type of systematic surveillance required to support such assertions would be extremely expensive and likely not justifiable. Therefore, we suggest that detection methods should be further refined and developed to ensure the systematic characterisation of reassortant viral lineages within large sets of genomic data. Furthermore, a public repository of known reassortant viruses should be established, enabling easy and direct comparisons between recent reassortant viral linages and other circulating viral lineages across a range of host types (perhaps something comparable to BLAST specifically for influenza reassortants). We would encourage additional genotyping and sequencing of endemic and locally transmitted viruses allowing for more detailed characterisation and differences between reassortant and non-reassortant viruses in the future. Lastly, we suggest that future laboratory experiments should be conducted to gain insight into the phenotypic and clinical changes that may arise as a consequence of reassortment.

## Additional files

Additional file 1: Figure S1. Number of papers published by year on reassortment, analysing all eight influenza A gene segments. * indicates data available for a partial year. **Figure S2.** Flow diagram showing the criteria for papers being included in the review. The number of papers excluded and included at each stage are indicated on the flow diagram. **Figure S3.** Inter-subtype reassortant analysis: Odds ratios for reporting inter-subtype reassortants by region, host and year. Crude (red) and adjusted (blue) odds ratios for the rate of reporting novel inter-subtype reassortants by region (a), host (b) and year (c). The dashed line at 1.00 on (a) and (b) represents the baseline level of risk, vertical bars crossing the dashed line indicate no effect at the 5 % level. The vertical lines above and below each point indicate 95 % confidence intervals. Adjusted odds are from the general additive logistic model of region and host as factors and year of isolated as a smooth term (c) [35]. Blue areas of (c) show 95 % confidence interval for smooth term for the contribution to the odds from the year of isolation. The univariate analysis where year was treated as a categorical variable is presented in Additional file 1: Table S1. Blue areas of c show 95 % confidence interval for smooth term for year of isolation. **Figure S4.** Intra-subtype reassortant analysis: Odds ratios for reporting intra-subtype reassortants by region, host and year. Crude (red) and adjusted (blue) odds ratios for the rate of reporting novel intra-subtype reassortants by region (a), host (b) and year (c). The dashed line at 1.00 on (a) and (b) represents the baseline level of risk, vertical bars crossing the dashed line indicate no effect at the 5 % level. The vertical lines above and below each point indicate 95 % confidence intervals. Adjusted odds are from the general additive logistic model of region and host as factors and year of isolated as a smooth term (c) [35]. Blue areas of (c) show 95 % confidence interval for smooth term for the contribution to the odds from the year of isolation. The univariate analysis where year was treated as a categorical variable is presented in Additional file 1: Table S1. Blue areas of c show 95 % confidence interval for smooth term for year of isolation. **Figure S5.** Funnel plot of the log odds of reporting reassorted viruses in China and Swine. A funnel plot of the log odds of identifying reassorted viruses in (a) swine hosts and (b) China across all years analysed in this study. Years are indicated on the plot. Group one includes the following years – 1991, 1992, 1993, 1994, 1995. Group two includes years 1996, 1997, 1998, 1999, 2000. Group 3 includes years 2011, 2012, and 2013. Banding of years was performed due to the low number of sequences uploaded to GenBank and reassortants identified in the years prior to 2000. The black dashed line indicates 0.00, the no effect line. The red dashed line indicates the estimated log odds from the adjusted regression model. Note that the data point for China in 2002 is not presented in (b) as the standard error was substantially larger than all other year groups analysed, therefore for visual reasons it has not been plotted. **Figure S6.** Odds ratios for reporting inter and intra-subtype reassortants for the sensitivity analysis for adding missed FRIs, by region, host and year. Crude (red) and adjusted (blue) odds ratios for the rate of reporting novel inter and intra-subtype reassortants for the sensitivity analysis by region (a), host (b) and year (c). The dashed line at 1.00 on (a) and (b) represents the baseline level of risk, vertical bars crossing the dashed line indicate no effect at the 5 % level. The vertical lines above and below each point indicate 95 % confidence intervals. Adjusted odds are from the general additive logistic model of region and host as factors and year of isolated as a smooth term (c) [35]. Blue areas of (c) show 95 % confidence interval for smooth term for the contribution to the odds from the year of isolation. The univariate analysis where year was treated as a categorical variable is presented in Additional file 4: Table S5. Blue areas of c show 95 % confidence interval for smooth term for year of isolation. (PDF 521 kb)

Additional file 2: **PRISMA 2009 Checklist. **(PDF 222 kb)

Additional file 3: **Review protocol.** (PDF 462 kb)

Additional file 4: **Database S1.** (XLSX 127 kb)

Additional file 5: **Regression analysis without highly homologous isolates removed.** (PDF 171 kb)

Additional file 6: **Sensitivity analysis to regression results.** (PDF 422 kb)

Additional file 7: **Labelled phylogenies.** (PDF 414 kb)

## Abbreviations

FRI: first reported isolate
MRCA: most recent common ancestor
SSRS: same sized random subset
