# Supplementary material for: A systematic review of reported reassortant viral lineages of influenza A
Source: BMC Infect Dis. 2016 Jan 5;16:3. doi: 10.1186/s12879-015-1298-9 (PMC4702296; doi:10.1186/s12879-015-1298-9)
Supplement: Supplementary file 1 — Number of papers published by year on reassortment, analysing all eight influenza A gene segments. * indicates data available for a partial year. Figure S2. Flow diagram showing the criteria for papers being included in the review. The number of papers excluded and included at each stage are indicated on the flow diagram. Figure S3. Inter-subtype reassortant analysis: Odds ratios for reporting inter-subtype reassortants by region, host and year. Crude (red) and adjusted (blue) odds ratios for the rate of reporting novel inter-subtype reassortants by region (a), host (b) and year (c). The dashed line at 1.00 on (a) and (b) represents the baseline level of risk, vertical bars crossing the dashed line indicate no effect at the 5 % level. The vertical lines above and below each point indicate 95 % confidence intervals. Adjusted odds are from the general additive logistic model of region and host as factors and year of isolated as a smooth term (c) [35]. Blue areas of (c) show 95 % confidence interval for smooth term for the contribution to the odds from the year of isolation. The univariate analysis where year was treated as a categorical variable is presented in Additional file 1: Table S1. Blue areas of c show 95 % confidence interval for smooth term for year of isolation. Figure S4. Intra-subtype reassortant analysis: Odds ratios for reporting intra-subtype reassortants by region, host and year. Crude (red) and adjusted (blue) odds ratios for the rate of reporting novel intra-subtype reassortants by region (a), host (b) and year (c). The dashed line at 1.00 on (a) and (b) represents the baseline level of risk, vertical bars crossing the dashed line indicate no effect at the 5 % level. The vertical lines above and below each point indicate 95 % confidence intervals. Adjusted odds are from the general additive logistic model of region and host as factors and year of isolated as a smooth term (c) [35]. Blue areas of (c) show 95 % confidence interval for smooth term for [file 12879_2015_1298_MOESM1_ESM.pdf]

Figure-S1 Pinsent

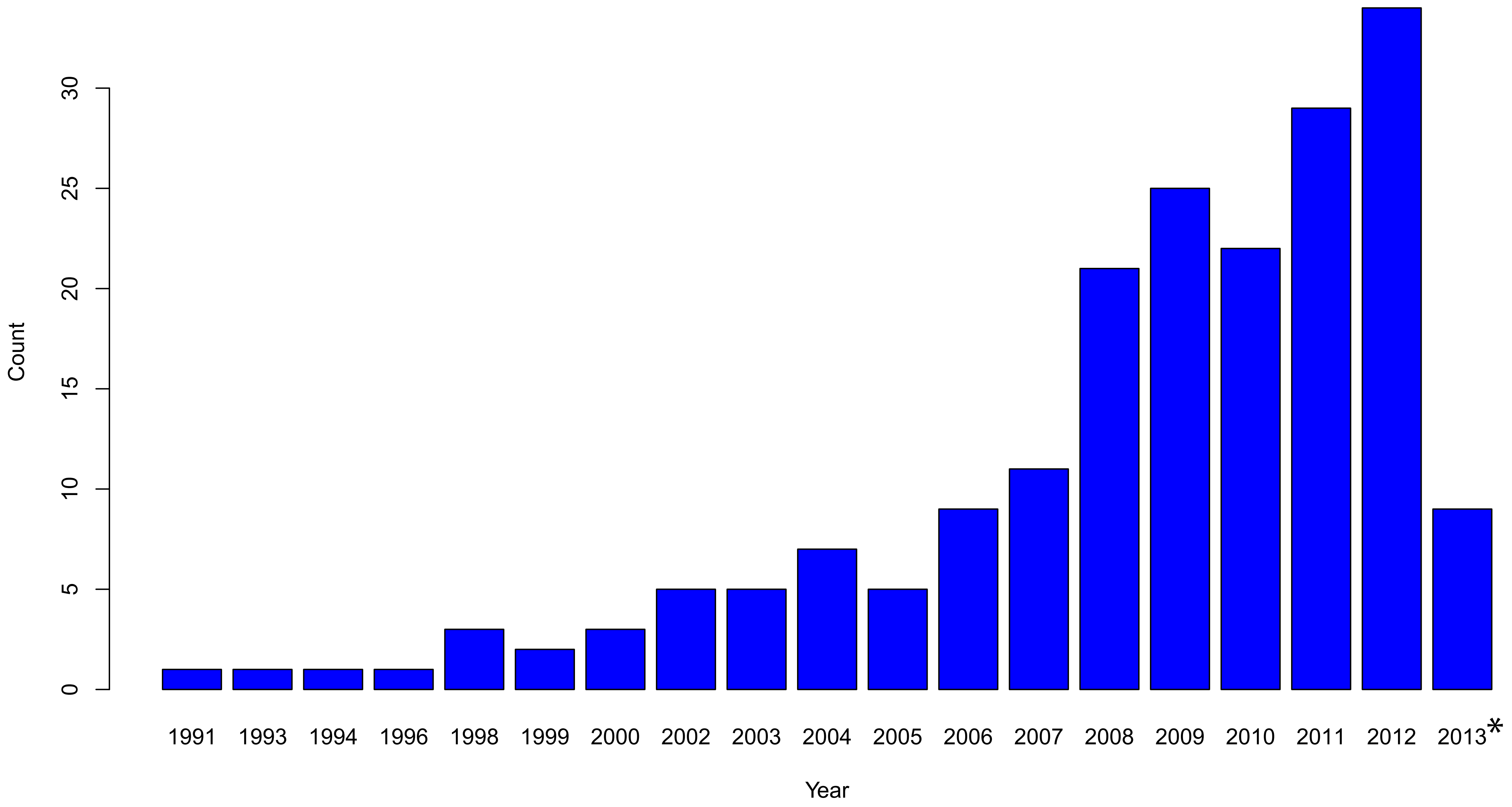

Figure-S2 Pinsent

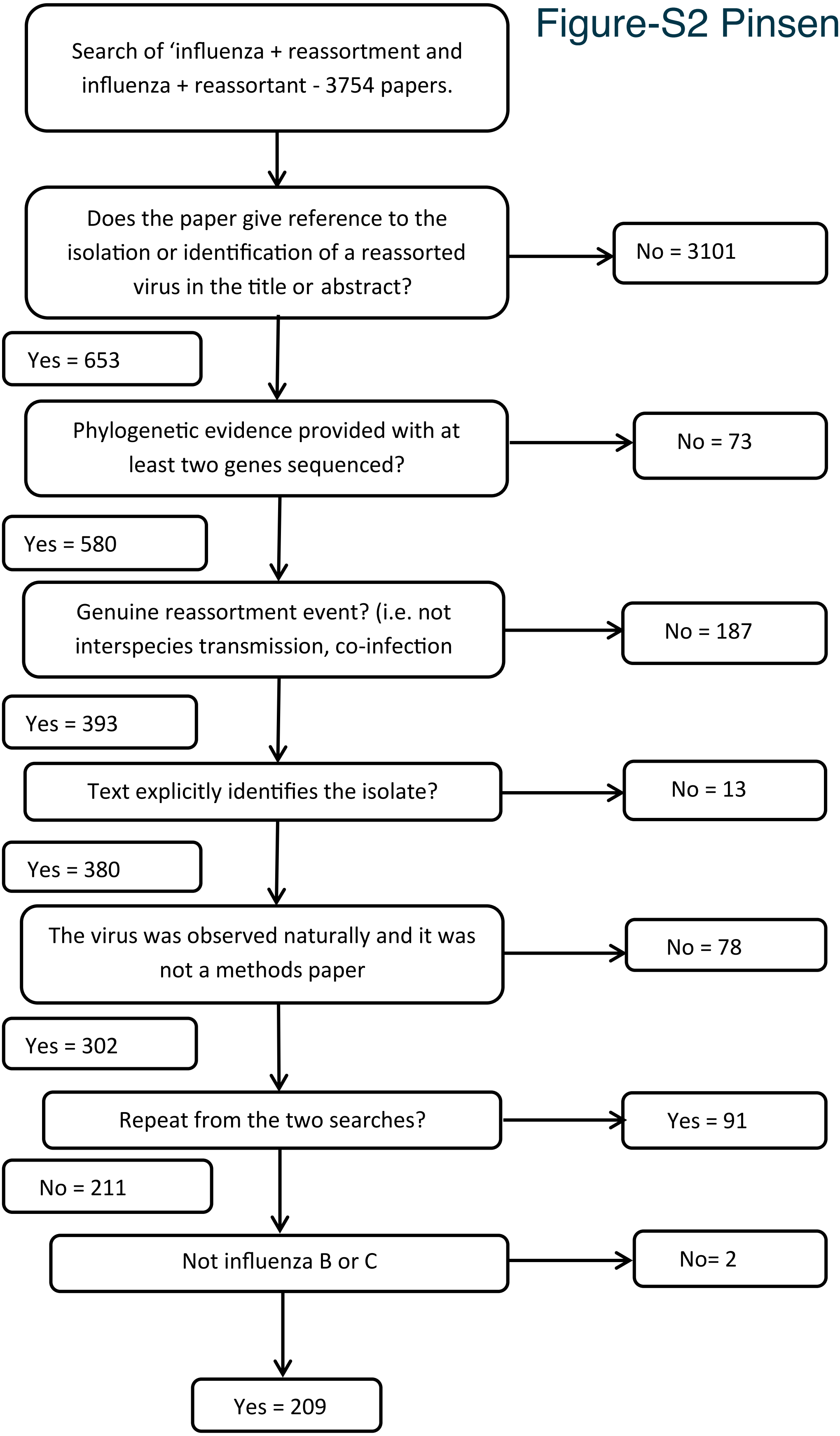

Figure-S3 Pinsent

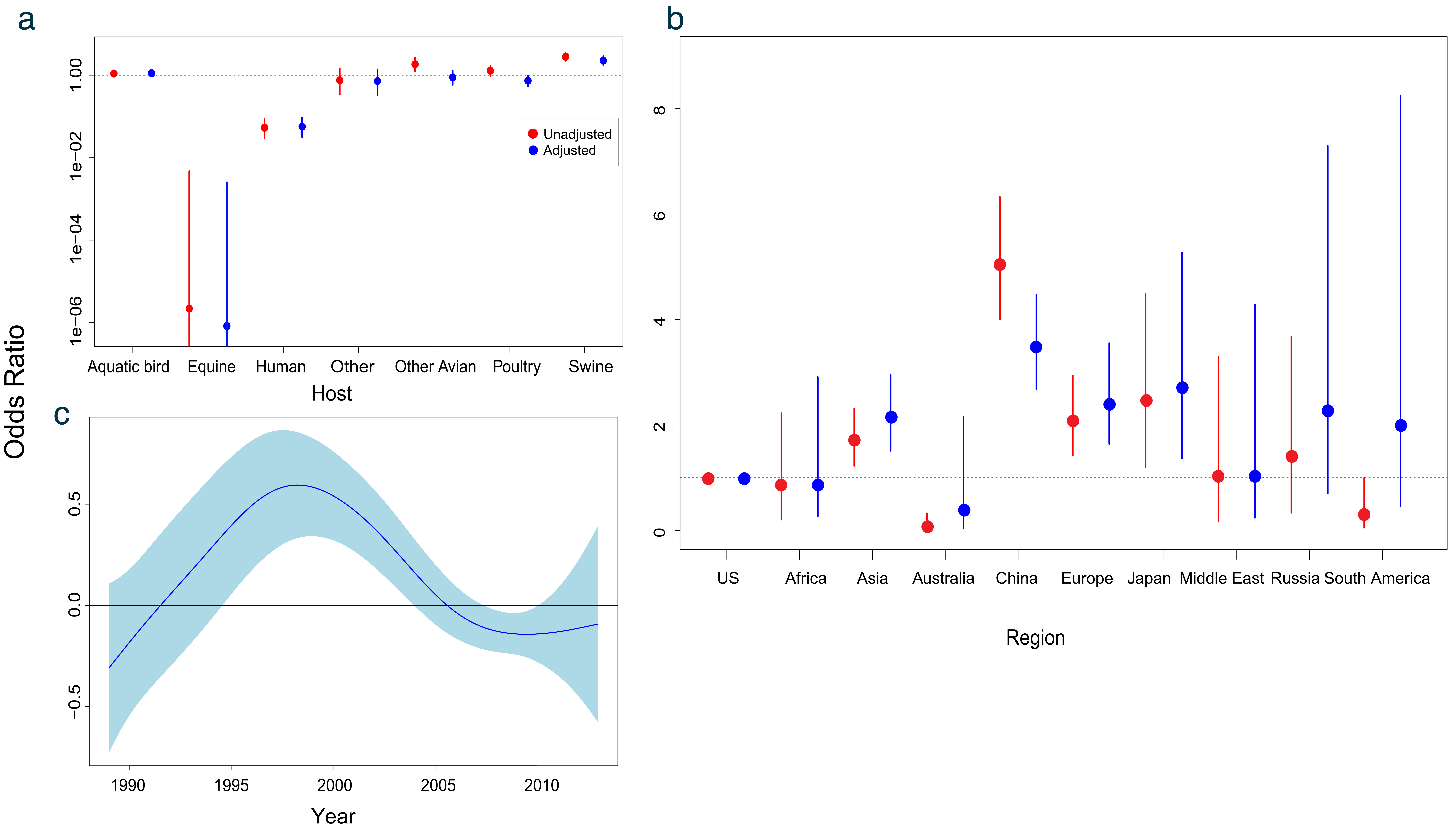

Figure-S4 Pinsent

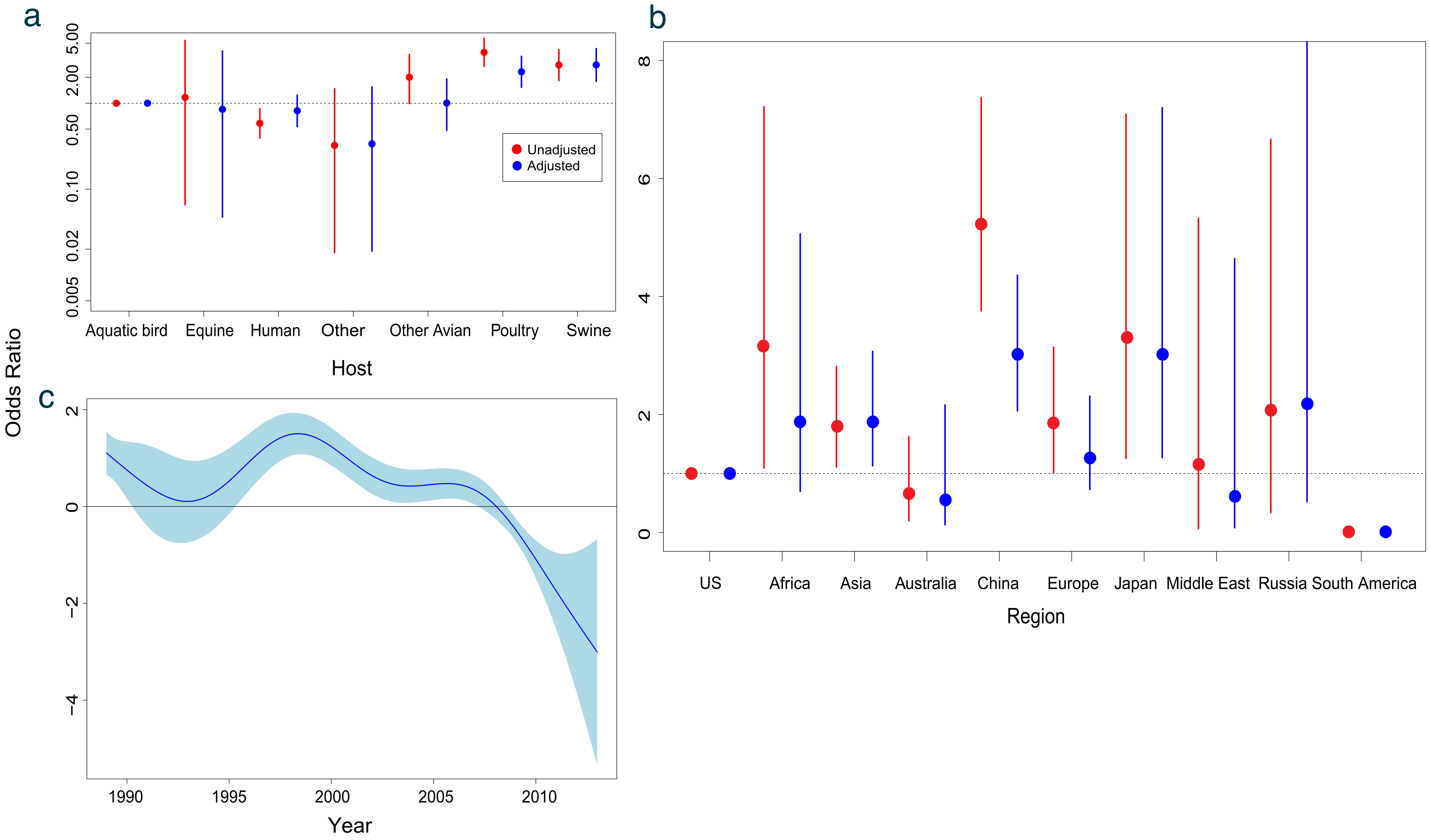

Figure S5 Pinsent

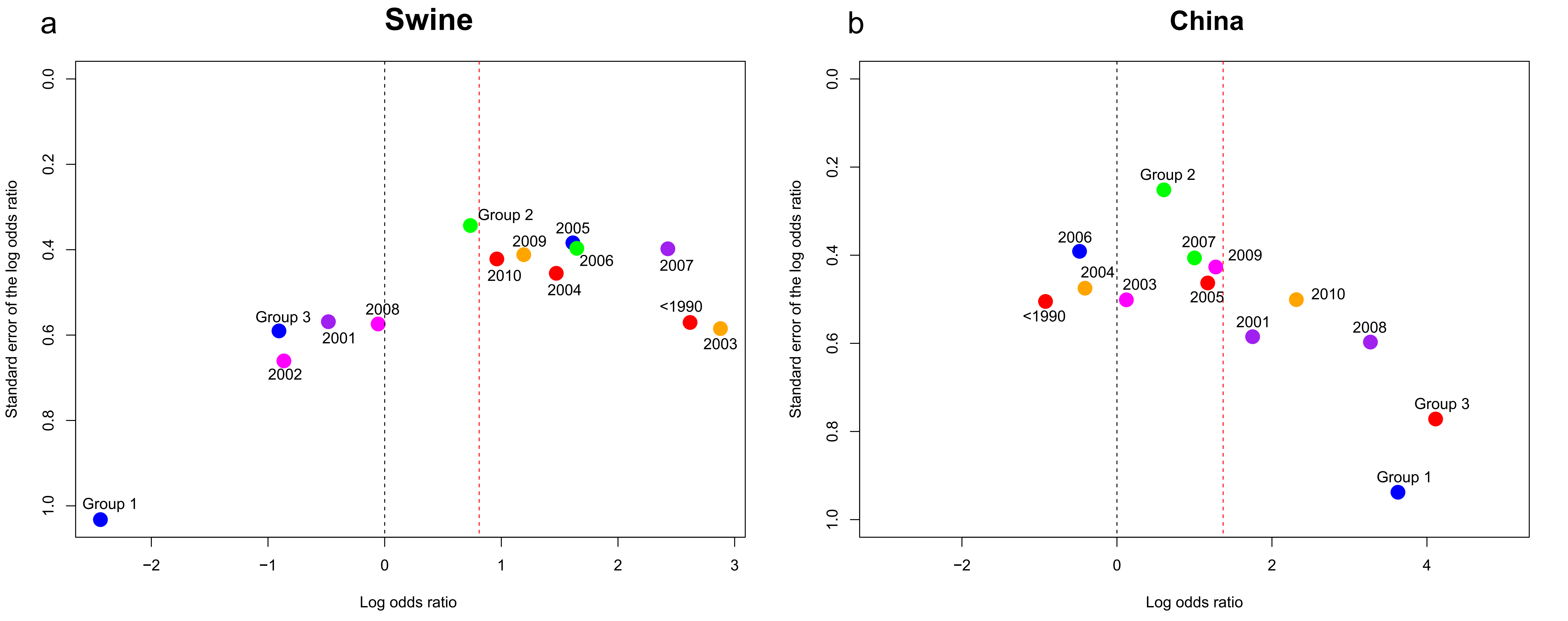

Table S1: Table for the univariate and multivariate odds for the general additive model analysis, for the 646 reported inter and intra-subtype reassortants.

	Odds Ratio (95% confidence interval) [2]	*p* [3]	Adjusted odds ratio (95% confidence interval) [4]	*p* [5]
**Host** [1]				
Aquatic bird	1		1	
Equine	0.25 (0.01 – 1.16)		0.23 (0.03 – 1.73)	
Human	0.17 (0.12 – 0.22)	*<0.001*	0.19 (0.14 – 0.25)	*<0.001*
Other Avian	1.90 (1.24 – 2.62)	*<0.001*	0.89 (0.63 – 1.26)	
Other	0.65 (0.30 – 1.21)		0.63 (0.32 – 1.25)	
Poultry	1.91 (1.52 – 2.40)	*<0.001*	1.07 (0.83 – 1.37)	
Swine	2.87 (2.34 – 3.52)	*<0.001*	2.48 (1.99 – 3.09)	*<0.001*
**Region**				
Africa	1.55 (0.69 – 2.98)		1.44 (0.68 – 3.04)	
Asia	1.73 (1.32– 2.24)	*<0.001*	2.07 (1.56 – 2.75)	
Australia	0.25 (0.08 – 0.55)	*<0.01*	0.51 (0.20 – 1.27)	
China	5.24 (4.33 – 6.36)	*<0.001*	3.45 (2.78 – 4.28)	*<0.001*
Europe	2.01 (1.46 – 2.71)	*<0.001*	1.92 (1.38 – 2.65)	*<0.001*
Japan	2.74 (1.56 – 4.49)	*<0.001*	2.80 (1.62 – 4.84)	*<0.001*
Middle East	1.07 (0.26 – 2.86)		0.88 (0.27 – 2.84)	
Russia	1.59 (0.56 – 3.53)		2.25 (0.90 – 5.64)	
South America	0.21 (0.03 – 0.68)	*<0.05*	0.78 (0.19 – 3.24)	
US	1		1	
**Year**				
<1990	1			
1990*	0 (0 – 0)			
1991	0.88 (0.21 – 2.48)			
1992	1.54 (0.45 – 3.94)			
1993	0.69 (0.12 – 1.74)			
1994	0.27 (0.04 – 0.88)	*<0.1*		
1995	0.36 (0.04 – 1.00)			
1996	0.84 (0.29 – 1.97)			
1997	3.70 (2.11 – 6.31)	*<0.001*		
1998	2.70 (1.49 – 4.70)	*<0.001*		
1999	1.79 (1.06 – 2.95)	*<0.05*		
2000	2.10 (1.34 – 3.26)	*<0.001*		
2001	1.69 (1.06 – 2.67)	*<0.05*		
2002	1.86 (1.20 – 2.86)	*<0.01*		
2003	1.33 (0.86 – 2.05)			
2004	1.21 (0.78 – 1.87)			
2005	1.06 (0.70 – 1.59)			
2006	1.16 (0.78 – 1.74)			
2007	0.74 (0.49 – 1.11)			
2008	0.71 (0.48 – 1.21)			
2009	0.24 (0.16 – 0.37)	*<0.001*		
2010	0.63 (0.41 – 0.98)	*<0.05*		
2011	0.64 (0.38 – 1.05)	*<0.1*		
2012	0.92 (0.47 – 1.68)			
2013	1.06 (0.05 – 5.20)			

Footnote: [1] The covariate and each level that was analysed when the analysis was performed. [2] The calculated unadjusted odds ratio for reporting inter- and intra-subtype reassortants for each covariate. Baseline levels for host, region of isolation and year are indicated in the table. Numbers in brackets indicate confidence intervals at the 95% level. [3] The p-value for each covariate for the unadjusted odds of reporting reassortants, calculated at the 95% significance level. [4] The calculated adjusted odds ratio for reporting inter- and intra-subtype reassortants for each covariate. Baseline levels for host, region of isolation and year are indicated in the table.

Numbers in brackets indicate confidence intervals at the 95% level. [5] The p-value for each covariate for the adjusted odds of reporting reassortants, calculated at the 95% significance level. We present the results for the univariate analysis for year, where year was treated as a categorical variable. Covaraites which have an * next to them have an estimated OR of 0, as no data on reported reassortants was identified for them.

Table S2: Table for the univariate and multivariate odds for the general additive model analysis, for the 416 reported inter-subtype reassortants.

	Odds Ratio (95% confidence interval) [2]	*p* [3]	Adjusted odds ratio (95% confidence interval) [4]	*p* [5]
**Host** [1]				
Aquatic bird	1		1	
Equine*	0 (0 – 0)		0 (0 – 0)	
Human	0.05 (0.03 – 0.08)	*<0.001*	0.05 (0.03 – 0.09)	*<0.001*
Other Avian	1.84 (1.25 – 2.65)	*<0.01*	0.89 (0.60 – 1.33)	
Other	0.75 (0.34 – 1.45)		0.70 (0.34 – 1.45)	
Poultry	1.29 (0.96 – 1.71)	*<0.1*	0.76 (0.52 – 1.04)	*<0.1*
Swine	2.81 (2.23 – 3.53)	*<0.001*	2.28 (1.78 – 2.92)	*<0.001*
**Region**				
Africa	0.83 (0.20 – 2.22)		0.89 (0.27 – 2.91)	
Asia	1.69 (1.22 – 2.31)	*<0.01*	2.11 (1.51 – 2.95)	*<0.001*
Australia	0.07 (0.004 – 0.32)	*<0.01*	0.29 (0.04 – 2.16)	
China	5.01 (4.00 – 6.32)	*<0.001*	3.46 (2.68 – 4.47)	*<0.001*
Europe	2.06 (1.42 – 2.93)	*<0.001*	2.41 (1.64 – 3.55)	*<0.001*
Japan	2.45 (1.19 – 4.48)	*<0.01*	2.69 (1.37 – 5.27)	*<0.01*
Middle East	1.03 (0.17 – 3.29)		1.02 (0.24 – 4.28)	
Russia	1.37 (0.33 – 3.67)		2.26 (0.70 – 7.29)	
South America	0.31 (0.05 – 0.99)		1.95 (0.46 – 8.24)	
US	1		1	
**Year**				
<1990	1			
1990*	0 (0 – 0)			
1991	1.41 (0.22 – 4.97)			
1992	2.76 (0.63 – 8.34)			
1993	1.25 (0.29 – 3.73)			
1994	0.65 (0.10 – 2.28)			
1995	0.36 (0.04 – 1.79)			
1996	1.21 (0.28 – 3.61)			
1997	4.40 (1.99 – 9.27)	*<0.001*		
1998	4.60 (2.18 – 9.40)	*<0.001*		
1999	2.10 (0.98 – 4.31)	*<0.05*		
2000	2.90 (1.57 – 5.43)	*<0.001*		
2001	2.72 (1.47 – 5.09)	*<0.01*		
2002	2.93 (1.63 – 5.37)	*<0.001*		
2003	2.65 (1.52 – 4.77)	*<0.001*		
2004	1.80 (0.98 – 3.33)			
2005	1.52 (0.86 – 2.75)			
2006	2.00 (1.17 – 3.54)	<0.05		
2007	1.05 (0.59 – 1.91)			
2008	1.30 (0.71 – 2.42)			
2009	0.42 (0.24 – 0.76)	*<0.01*		
2010	1.39 (0.80 – 2.47)			
2011	1.42 (0.76 – 2.66)			
2012	2.24 (1.07 – 4.55)	*<0.05*		
2013	2.57 (0.14 – 0.13)			

Footnote: [1] The covariate and each level that was analysed when the analysis was performed. [2] The calculated unadjusted odds ratio for reporting inter-subtype reassortants for each covariate. Baseline levels for host, region of isolation and year are indicated in the table. Numbers in brackets indicate confidence intervals at the 95% level. [3] The p-value for each covariate for the unadjusted odds of reporting reassortants, calculated at the 95% significance level. [4] The calculated adjusted odds ratio for reporting inter-subtype reassortants for each covariate. Baseline levels for host, region of isolation and year are indicated in the table. Numbers in brackets

indicate confidence intervals at the 95% level. [5] The p-value for each covariate for the adjusted odds of reporting reassortants, calculated at the 95% significance level. We present the results for the univariate analysis for year, where year was treated as a categorical variable. Covaraites which have an * next to them have an estimated OR of 0, as no data on reported reassortants was identified for them.

Table S3: Table for the univariate and multivariate odds for the general additive model analysis, for the 230 reported intra-subtype reassortants.

	Odds Ratio (95% confidence interval) [2]	*p* [3]	Adjusted odds ratio (95% confidence interval) [4]	*p* [5]
**Host** [1]				
Aquatic bird	1		1	
Equine	1.16 (0.06 – 5.39)		0.87 (0.11 – 6.50)	
Human	0.58 (0.39 – 0.86)	*<0.01*	0.77 (0.51 – 1.17)	
Other Avian	2.00 (0.99 – 3.70)	*<0.05*	0.98 (0.49 – 1.94)	
Other	0.32 (0.01 – 1.47)		0.34 (0.04 – 2.51)	
Poultry	3.91 (2.67 – 5.71)	*<0.001*	2.28 (1.51 – 3.44)	*<0.001*
Swine	2.78 (1.83 – 4.19)	*<0.001*	2.64 (1.70 – 4.08)	*<0.001*
**Region**				
Africa	3.15 (1.09 – 7.21)	*<0.05*	1.89 (0.70 – 5.06)	
Asia	1.78 (1.10 – 2.81)	*<0.05*	1.86 (1.13–3.07)	
Australia	0.66 (0.20 – 1.62)		0.53 (0.13 – 2.16)	
China	5.23 (3.75 – 7.36)	*<0.001*	3.00 (2.06–4.36)	*<0.001*
Europe	1.83 (1.01 – 3.14)	*<0.05*	1.30 (0.73 – 2.31)	
Japan	3.27 (1.25 – 7.08)	*<0.01*	3.02 (1.27–7.20)	<0.05
Middle East	1.16 (0.06 – 5.32)		0.62 (0.08 – 4.64)	
Russia	2.05 (0.33 – 6.66)		2.19 (0.52–9.15)	
South America	0 (0 – 0)		0 (0 – 0)	
US	1		1	
**Year**				
<1990	1			
1990*	0 (0 – 0)			
1991	0.50 (0.02 – 2.43)			
1992	0.65 (0.03 – 3.12)			
1993	0.30 (0.01 – 1.42)			
1994	0 (0 – 0)			
1995	0.26 (0.01 – 1.27)			
1996	0.58 (0.09 – 1.98)			
1997	2.88 (1.30 – 5.92)	*<0.01*		
1998	1.23 (0.41 – 3.00)			
1999	1.52 (0.73 – 3.00)			
2000	1.44 (0.75 – 2.70)			
2001	0.92 (0.43 – 1.83)			
2002	1.04 (0.52 – 1.97)			
2003	0.38 (0.15 – 0.84)	*<0.001*		
2004	0.77 (0.39 – 1.45)			
2005	0.72 (0.40 – 1.28)			
2006	0.55 (0.29 – 1.03)	*<0.1*		
2007	0.51 (0.28 – 0.92)	*<0.05*		
2008	0.39 (0.17 – 0.78)	*<0.05*		
2009	0.12 (0.06 – 0.23)	*<0.001*		
2010	0.10 (0.03 – 0.27)	*<0.001*		
2011	0.09 (0.01 – 0.30)	*<0.01*		
2012	0 (0 – 0)			
2013	0 (0 – 0)			

Footnote: [1] The covariate and each level that was analysed when the analysis was performed. [2] The calculated unadjusted odds ratio for reporting intra-subtype reassortants for each covariate. Baseline levels for host, region of isolation and year are indicated in the table. Numbers in brackets indicate confidence intervals at the 95% level. [3] The p-value for each covariate for the unadjusted odds of reporting reassortants, calculated at the 95% significance level. [4] The calculated adjusted odds ratio for reporting intra-subtype reassortants for each covariate. Baseline levels for host, region of isolation and year are indicated in the table. Numbers in brackets

indicate confidence intervals at the 95% level. [5] The p-value for each covariate for the adjusted odds of reporting reassortants, calculated at the 95% significance level. We present the results for the univariate analysis for year, where year was treated as a categorical variable. Covaraites which have an * next to them have an estimated OR of 0, as no data on reported reassortants was identified for them.
