# Supplementary material for: A systematic review of reported reassortant viral lineages of influenza A
Source: BMC Infect Dis. 2016 Jan 5;16:3. doi: 10.1186/s12879-015-1298-9 (PMC4702296; doi:10.1186/s12879-015-1298-9)
Supplement: Supplementary file 3 — Review protocol. (PDF 462 kb) [file 12879_2015_1298_MOESM3_ESM.pdf]

A Systematic Review of Reported Reassortant Viral Lineages of Influenza A

Amy Pinsent [*1], Christophe Fraser [1], Neil M. Ferguson [1], Steven Riley [1]

1. MRC Centre for Outbreak Analyses and Modelling, Department of Infectious Disease Epidemiology, School of Public Health, Imperial College London.

*amy.pinsent07@imperial.ac.uk

Protocol for the systematic review and meta-analysis on reassortant viral lineages of influenza A

1. Reviewer

Amy Pinsent [1]

1. MRC Centre for Outbreak Analyses and Modelling, Department of Infectious Disease Epidemiology, School of Public Health, Imperial College London.

2. Review purpose

The study aims to collate and analyse existing information on reassortant viral lineages of influenza A to identify risk factors for the emergence of reassortant viral lineages and whether there are any genetic differences between reassorted data and same size random samples from GenBank for the same host/subtype combination.

3. Introduction

Although the importance of reassortment as a mechanism for driving the emergence of novel influenza genotypes with pandemic potential has been recognized for many years (1-3), an understanding of this process at the population level has, until recently, been impossible because of a lack of genomic data. However, the increasingly widespread

availability of whole-genome sequencing (4) has permitted a rapid expansion (Figure S1) of high quality descriptive studies that rely on genomic data. Pathogen-dynamic studies of reassortment have previously focussed on specific influenza subtypes (5-7), hosts (4, 8, 9) or evolutionary events (3, 10, 11). More recently work has estimated rates of reassortment within a particular viral lineage (12) and to identify high-risk areas in which reassortment may occur (13). However, broader descriptions of patterns of observed reassortment remain lacking. We examined the extent to which theories of geographical (1) and host (14, 15) drivers of pandemic emergence may or may not be reflected in the frequency with which novel reassortants have been identified.

4. Search strategy and study selection

We will conduct electronic searches in PubMed (MEDLINE) and Web of Knowledge (all databases) to identify relevant articles. There is no restriction regarding language, publication period or study design.

Search terms:

- PubMed and Web of Knowledge: "influenza AND reassortment" or "influenza AND reassortant"

All titles and abstracts were scanned, if the title was not rejected then the full text of the article was obtained and carefully reviewed for inclusion by (AP). Inclusion or exclusion of a study was evaluated based on the inclusion/exclusion criteria but was specific to clear phylogenetic evidence for the occurrence of reassortment. If unsure of the inclusion of any articles SR was consulted.

5. Inclusion criteria and Exclusion criteria

Inclusion criteria

- First report of a novel reassortant viral lineages of influenza A. This could be any subtype and any host.

- Presentation of clear phylogenetic evidence and suggestion that greater than 2 sequences had been deposited in GenBank. The presence of all 8 genes in GenBank was later checked.

Exclusion criteria

- Sequences not deposited in GenBank. i.e published in GISAID or not at all.
- At least 2 genes sequenced shown in the article.
- Repeat isolate from a previous paper eg. Novel 2013 H7N9 or H1N1 2009 pandemic isolates.
- No explicit identification of isolates that had undergone reassortment.
- Laboratory studies of reassortant isolates i.e non-natural strains or ones that were being developed as vaccine strains.
- Articles which highlighted the occurrence of co-infection or interspecies transmission of virus that did not result in reassortment.
- Articles that focused on the algorithmic detection of viruses to classify them as reassortant were not included.

6. Data extraction

Data extraction was performed by AP. Data was recorded in an excel file. All whole genomes identified are in Database S1.

Extraction of study characteristics and reassortment data

- First author
- Year of publication
- Title of paper
- Journal published in
- Name of the isolate or isolates that were reported as reassortant
- Host type from which the isolate was collected
- Year isolate was collected
- Geographic region the isolate was collected

- Accession numbers for the genes for each isolate

Selection of data for the meta-analysis

- We defined reassortment to be the viral exchange of one whole gene segment, between two different viral lineages, such that at least one gene from the viral isolate was located dis-concordantly to the other 7 genes on a phylogenetic tree. This criterion ensured clear detection of reassortant strains.
- After a strain duplication algorithm was applied to the isolates for which data of all genes was available, the resulting set of isolates were used in the meta-analysis.
- We downloaded all the available whole genomes from Genbank and used this data as the denominator for the general additive model.
- Reassortant data was stratified according to year of isolation, host type and region of isolation. The odds of identifying a reassortant virus out of this data set was then computed in R.

Selection of data for the comparative trees and hamming distance distributions

- The denominator gathered from GenBank was used to draw the same size random samples for each host/subtype combination. Data was subset according to host and subtype.
- From this subset for a specific host subtype combination the same number of random samples were drawn as there were for that host/subtype combination in the final reassorted data set. 10 random samples for each host/subtype combination were drawn.

7. Risk of bias

Genetic similarity between isolates for which whole genome data was available was assessed. A filtering algorithm was applied to remove duplicate isolates based on gene specific sequence homology. If isolates were very genetically similar it was checked to see if they were reported in the same article as the same reassortant, if they were the more recent isolate was removed, if they were reported in different articles an alternative route was taken. See Figure 1 for further detail.

We used the same criteria to classify reassortant virus across all papers examined ensuring consistency. By subsequently restricting the analysis to whole genomes we reduced the chances that reassortment may have occurred on other genes but gone unnoticed by the author. Quality of the sequences themselves was not assessed, nor the robustness of the trees on which the reassortant report was made as we were interested in capturing as many reports as possible.

8. Statistical model for the probability of reassortment

Regression analysis is performed for each of the 3 covariates available, with all the full genomes available in GenBank for all years, host types and regions as the denominator data. The univariate logistic regression analysis in R (16) and the odds ratio (OR) for each of the covariates for the identification of a reassortant isolate being identified can be calculated. The effect of covariates is considered significant when the p-value is <0.05 or its 95%CI is not overlapped with the original one.

To examine the relationship between: host, geographic region and year of isolation; and the probability that a given publically available genome was an FRI we used a multivariate general additive model. To compare between models we used the Akaike Information Criterion (AIC), which gives the likelihood of the model minus the number of parameters within the model. The addition of each of the covariates significantly improved the AIC score. We fitted a smoothing spline to year as a covariate. This model was developed using the mgcv package in R (16, 17)

References

1. Webster RG, Laver WG, Air GM, Schild GC. Molecular mechanisms of variation in influenza viruses. Nature. 1982;296(5853):115-21.
2. Belshe RB. The origins of pandemic influenza - Lessons from the 1918 virus. N Engl J Med. 2005 Nov;353(21):2209-11.
3. Smith GJD, Vijaykrishna D, Bahl J, Lycett SJ, Worobey M, Pybus OG, et al. Origins and evolutionary genomics of the 2009 swine-origin H1N1 influenza A epidemic. Nature. 2009 Jun;459(7250):1122-U107.

4. Holmes EC, Ghedin E, Miller N, Taylor J, Bao YM, St George K, et al. Whole-genome analysis of human influenza A virus reveals multiple persistent lineages and reassortment among recent H3N2 viruses. PLoS Biol. 2005 Sep;3(9):1579-89.
5. Li CJ, Yu KZ, Tian GB, Yu DD, Liu LL, Jing B, et al. Evolution of H9N2 influenza viruses from domestic poultry in Mainland China. Virology. 2005 Sep;340(1):70-83.
6. Vijaykrishna D, Poon LLM, Zhu HC, Ma SK, Li OTW, Cheung CL, et al. Reassortment of Pandemic H1N1/2009 Influenza A Virus in Swine. Science. 2010 Jun;328(5985):1529-.
7. Nelson MI, Viboud C, Simonsen L, Bennett RT, Griesemer SB, St George K, et al. Multiple reassortment events in the evolutionary history of H1N1 influenza A virus since 1918. PLoS pathogens. 2008 2008 Feb;4(2):e1000012.
8. Vijaykrishna D, Smith GJD, Pybus OG, Zhu HC, Bhatt S, Poon LLM, et al. Long-term evolution and transmission dynamics of swine influenza A virus. Nature. 2011 May;473(7348):519-U263.
9. Nelson MI, Detmer SE, Wentworth DE, Tan Y, Schwartzbard A, Halpin RA, et al. Genomic reassortment of influenza A virus in North American swine, 1998-2011. J Gen Virol. 2012 Dec;93:2584-9.
10. Nelson MI, Simonsen L, Viboud C, Miller MA, Holmes EC. The origin and global emergence of adamantane resistant A/H3N2 influenza viruses. Virology. 2009 Jun;388(2):270-8.
11. Smith GJD, Bahl J, Vijaykrishna D, Zhang JX, Poon LLM, Chen HL, et al. Dating the emergence of pandemic influenza viruses. Proc Natl Acad Sci U S A. 2009 Jul;106(28):11709-12.
12. Lycett SJ, Baillie G, Coulter E, Bhatt S, Kellam P, McCauley JW, et al. Estimating reassortment rates in co-circulating Eurasian swine influenza viruses. J Gen Virol. 2012 Nov;93:2326-36.
13. Fuller TL, Gilbert M, Martin V, Cappelle J, Hosseini P, Njabo KY, et al. Predicting hotspots for influenza virus reassortment. Emerging infectious diseases. 2013 2013;19(4):581-8.
14. Scholtissek C. Pis as mixing vessels for the creation of new pandemic influenza A viruses. Medical Principles and Practice. 1990;2(2):65-71.
15. Thontiravong A, Kitikoon P, Wannaratana S, Tantilertcharoen R, Tuanudom R, Pakpinyo S, et al. Quail as a potential mixing vessel for the generation of new reassortant influenza A viruses. Vet Microbiol. 2012 Dec;160(3-4):305-13.
16. R Core Development Team. R: A Language and Environment for Statistical Computing. Vienna, Austria: R Foundation for Statistical Computing; 2013.
17. Wood S. mgcv: Mixed GAM Computation Vehicle with GCV/AIC/REML smoothness estimation. 2013 [cited; Available from: http://cran.r-project.org/web/packages/mgcv/index.html
