# Supplementary material for: A systematic review of reported reassortant viral lineages of influenza A
Source: BMC Infect Dis. 2016 Jan 5;16:3. doi: 10.1186/s12879-015-1298-9 (PMC4702296; doi:10.1186/s12879-015-1298-9)
Supplement: Supplementary file 5 — Regression analysis without highly homologous isolates removed. (PDF 171 kb) [file 12879_2015_1298_MOESM5_ESM.pdf]

Table S6: Table for the univariate and multivariate odds for the general additive model analysis, for the 731 FRIs initially identified in the review.

	Odds Ratio (95% confidence interval) [2]	*p* [3]	Adjusted odds ratio (95% confidence interval) [4]	*p* [5]
**Host** [1]				
Aquatic bird	1		1	
Equine	0.5 (0.08 –1.59)		0.39 (0.06 – 1.28)	
Human	0.25 (0.20 – 0.32)	*<0.001*	0.30 (0.23 – 0.39)	*<0.001*
Other Avian	1.96 (1.41 – 2.68)	*<0.001*	0.97 (0.68 – 1.35)	
Other	0.63 (0.29 – 1.16)		0.63 (0.29 – 1.18)	
Poultry	2.11 (1.69 – 2.61)	*<0.001*	1.21 (0.95 – 1.53)	
Swine	3.11 (2.55 – 3.78)	*<0.001*	2.73 (2.20 – 3.39)	*<0.001*
**Region**				
Africa	1.53 (0.72 – 2.84)		1.31 (0.60 – 2.53)	
Asia	1.78 (1.39 – 2.27)	*<0.001*	2.18 (1.67 – 2.83)	*<0.001*
Australia	0.35 (0.15 – 0.67)	*<0.01*	0.53 (0.23 – 1.04)	
China	5.00 (4.18 – 6.00)	*<0.001*	3.27 (2.66 – 4.03)	*<0.001*
Europe	2.14 (1.61 – 2.82)	*<0.001*	1.87 (1.37 – 2.52)	*<0.001*
Japan	2.71 (1.59 – 4.32)	*<0.001*	2.61 (1.50 – 4.29)	*<0.001*
Middle East	0.93 (0.23 – 2.49)		0.73 (0.17 – 2.01)	
Russia	1.38 (0.48 – 3.07)		1.96 (0.68 – 4.43)	
South America	0.28 (0.07 – 0.75)	*<0.05*	0.87 (0.21 – 2.36)	
US	1		1	
**Year**				
<1990	1			
1990	0.38 (0.02 – 1.81)			
1991	0.87 (0.26 – 2.17)			
1992	1.12 (0.33 – 2.82)			
1993	1.17 (0.53 – 2.30)			
1994	0.29 (0.07 – 0.81)	*<0.05*		
1995	0.22 (0.03 – 0.72)	*<0.05*		
1996	1.00 (0.43 – 2.04)			
1997	2.99 (1.76 – 4.91)	*<0.001*		
1998	2.59 (1.53 – 4.23)	*<0.001*		
1999	1.90 (1.21 – 2.92)	*<0.01*		
2000	1.70 (1.12 – 2.54)	*<0.05*		
2001	1.31 (0.85 – 1.99)			
2002	1.49 (1.00 – 2.21)	*<0.05*		
2003	1.02 (0.68 – 1.52)			
2004	0.97 (0.65 – 1.44)			
2005	0.80 (0.55 – 1.16)			
2006	0.89 (0.61 – 1.28)			
2007	0.59 (0.40 – 0.85)	*<0.01*		
2008	0.68 (0.45 – 1.06)			
2009	0.19 (0.13 – 0.28)	*<0.001*		
2010	0.48 (0.32 – 0.72)	*<0.001*		
2011	0.46 (0.28 – 0.74)	*<0.01*		
2012	0.67 (0.35 – 1.20)			
2013	0.77 (0.04 – 3.75)			

Footnote: [1] The covariate and each level that was analysed when the analysis was performed. [2] The calculated unadjusted odds ratio for reporting inter-subtype reassortants for each covariate. Baseline levels for host, region of isolation and year are indicated in the table. Numbers in brackets indicate confidence intervals at the 95% level. [3] The p-value for each covariate for the unadjusted odds of reporting reassortants, calculated at the 95%

significance level. [4] The calculated adjusted odds ratio for reporting inter-subtype reassortants for each covariate. Baseline levels for host, region of isolation and year are indicated in the table. Numbers in brackets indicate confidence intervals at the 95% level. [5] The p-value for each covariate for the adjusted odds of reporting reassortants, calculated at the 95% significance level. We present the results for the univariate analysis for year, where year was treated as a categorical variable.
