# Supplementary material for: A systematic review of reported reassortant viral lineages of influenza A
Source: BMC Infect Dis. 2016 Jan 5;16:3. doi: 10.1186/s12879-015-1298-9 (PMC4702296; doi:10.1186/s12879-015-1298-9)

Additional file 6:

Sensitivity analysis to the regression results

13 papers were excluded in the initial review on the basis that they did not explicitly make available or provide the isolate name for isolates which they claimed to have identified as unique reassortant virus. Of these 13 articles, seven made reference to the possible identification of novel avian reassortants, across a broad range of subtypes. Four made reference to additional swine reassortants. While six made reference to possible additional human reassortants.

As much information as possible from each of the 13 articles was obtained on the possible additional number of reassortants, if available, the number of samples analysed in each study, host type, geographic region and the time span over which the isolates were assessed. As we did not know the exact details about isolates that may have been missed from these articles there was a chance the additional isolates added were repeat genotypes (genotype duplicates). This may be particularly true for isolates whose reassortants have been classified according to different letter lineages; this was most commonly done for H6N1 and H5N1 reassortants. A number of letter lineages were likely to have been already included as FRIs, where the same letter linage was provided it was assumed they were just different isolates that represented the same reassortant lineage.

An additional 125 possible FRIs were inferred from the 13 articles. By far the largest number of additional suspected FRIs were identified in aquatic birds in the USA. A breakdown of the number of FRIs added to the data by host and region is indicated in Table S4.

Table S4: The additional number of FRIs that were added to the 648 FRIs. These numbers were inferred from the 13 studies that were not included in the original analysis because they did not explicitly identify the isolates in the text.

	**Aquatic Bird**	**Poultry**	**Swine**	**Other**	**Human**
**Asia**	13	0	0	1	2
**Australia**	0	0	0	0	5
**China**	1	0	5	0	8
**Europe**	0	0	8	0	0
**USA**	67	2	0	0	5

The general additive models were reapplied to the new data set with the additional 125 possible FRIs. As with the previous data, the stepwise addition of each of the 3 covariates resulted in a significant improvement in the AIC score for the model. Once again, the odds of reporting reassortants from swine hosts was elevated above the baseline in both the uni and multivariate analysis: OR: 2.31 (95% CI: 1.91 –2.79) and OR: 2.14 (95% CI: 1.74 – 2.63), respectively (Table S5 and Figure S6 a). However, this effect was reduced in comparison to

the model that contained only 646 FRIs, this was most likely due to the high number of additional aquatic bird FRIs that were added to the data. Although despite the addition of 81 aquatic bird FRIs, the odds of reporting swine FRIs was still high. Despite the addition of 20 extra human FRIs the odds of reporting human FRIs was significantly reduced relative to the baseline, OR: 0.22 (95% CI: 0.17 – 0.28) (p-value *<0.001)* (Table S5 and Figure S6 b). The odds of reporting FRIs from Europe, China and Japan were all elevated above the baseline, however the effect size of the odds for these regions was reduced when compared to the model which contained 648 FRIs. Most likely as a result of the large number of FRIs added to USA, which was used as the baseline region. A spline was fitted to year as a covariate and as with the previous model the fit was statistically significant (Table S5 and Figure S6 c). Only one year (1997) in the univariate analysis was significantly above the baseline for having FRIs reported (OR: 2.53 (95% CI: 1.55 – 4.00).

Table S5: Table for the univariate and multivariate odds for the general additive model analysis, for the increased number of FRI isolates in the sensitivity analysis (771 isolates).

	Odds Ratio (95% confidence interval) [2]	*p* [3]	Adjusted odds ratio (95% confidence interval) [4]	*p* [5]
**Host** [1]				
Aquatic bird	1		1	
Equine	0.19 (0.01 – 0.85)		0.13 (0.00 – 0.63)	
Human	0.18 (0.14 – 0.23)	*<0.001*	0.22 (0.17 – 0.28)	*<0.001*
Other Avian	1.41 (1.00 – 1.92)	*<0.05*	0.55 (0.53 – 0.99)	
Other	0.53 (0.26 – 0.96)		0.76 (0.27 – 1.06)	
Poultry	1.44 (1.15 – 1.78)	*<0.001*	0.90 (0.71 – 1.14)	
Swine	2.31 (1.91 –2.79)	*<0.001*	2.14 (1.74 – 2.63)	*<0.001*
**Region**				
Africa	1.05 (0.47 – 2.00)		0.96 (0.42 – 1.90)	
Asia	1.38 (1.08– 1.74)	*<0.01*	1.78 (1.37 – 2.29)	*<0.001*
Australia	0.34 (0.16 – 0.61)	*<0.001*	0.54 (0.26 – 1.01)	
China	3.74 (3.15 – 4.43)	*<0.001*	2.55 (2.10 – 3.11)	*<0.001*
Europe	1.55 (1.17 – 2.04)	*<0.01*	1.49 (1.10 – 2.00)	<0.01
Japan	1.85 (1.06 – 3.00)	*<0.05*	1.87 (1.04 – 3.31)	*<0.05*
Middle East	0.72 (0.17 – 1.92)		0.65 (0.15 – 1.77)	
Russia	1.07 (0.37 – 2.37)		1.51 (0.52 – 3.42)	
South America	0.14 (0.02 – 0.45)	*<0.01*	0.54 (0.08 – 1.73)	
US	1		1	
**Year**				
<1990	1			
1990	0 (0 – 0)			
1991	0.47 (0.11 –1.29)			
1992	0.82 (0.24 – 2.05)			
1993	0.46 (0.16 – 1.06)			
1994	0.21 (0.05 – 0.59)	*<0.01*		
1995	0.16 (0.02 – 0.52)	*<0.01*		
1996	0.45 (0.15 – 1.02)			
1997	2.53 (1.55 – 4.00)	*<0.001*		
1998	1.80 (1.07 – 2.91)	*<0.05*		
1999	1.00 (1.00 – 1.56)			
2000	1.24 (0.84 – 1.81)			
2001	1.02 (0.68 – 1.50)			
2002	1.07 (0.73 – 1.54)			
2003	0.80 (0.55 – 1.16)			
2004	0.76 (0.52 – 1.10)			
2005	0.61 (0.43 – 0.86)	<0.01		
2006	0.81 (0.59 – 1.12)			
2007	0.47 (0.33 – 0.66)	*<0.001*		
2008	0.60 (0.41 – 0.85)	<0.01		
2009	0.14 (0.10 – 0.20)	*<0.001*		
2010	0.34 (0.23 – 0.49)	*<0.001*		
2011	0.34 (0.21 – 0.53)	*<0.001*		
2012	0.49 (0.26 – 0.86)	*<0.05*		
2013	0.56 (0.03 – 2.73)			

[1] The covariate and each level that was analysed when the analysis was performed. [2] The calculated unadjusted odds ratio for reporting inter-subtype reassortants for each covariate. Baseline levels for host, region of isolation and year are indicated in the table. Numbers in brackets indicate confidence intervals at the 95% level. [3] The p-value for each covariate for the unadjusted odds of reporting reassortants, calculated at the 95% significance level. [4] The calculated adjusted odds ratio for reporting inter-subtype reassortants for each covariate. Baseline

levels for host, region of isolation and year are indicated in the table. Numbers in brackets indicate confidence intervals at the 95% level. [5] The p-value for each covariate for the adjusted odds of reporting reassortants, calculated at the 95% significance level. We present the results for the univariate analysis for year, where year was treated as a categorical variable. An additional 125 FRIs were added to the 646 FRIs from papers that did not explicitly identify FRIs in the text.

Figure - S6 Pinsent

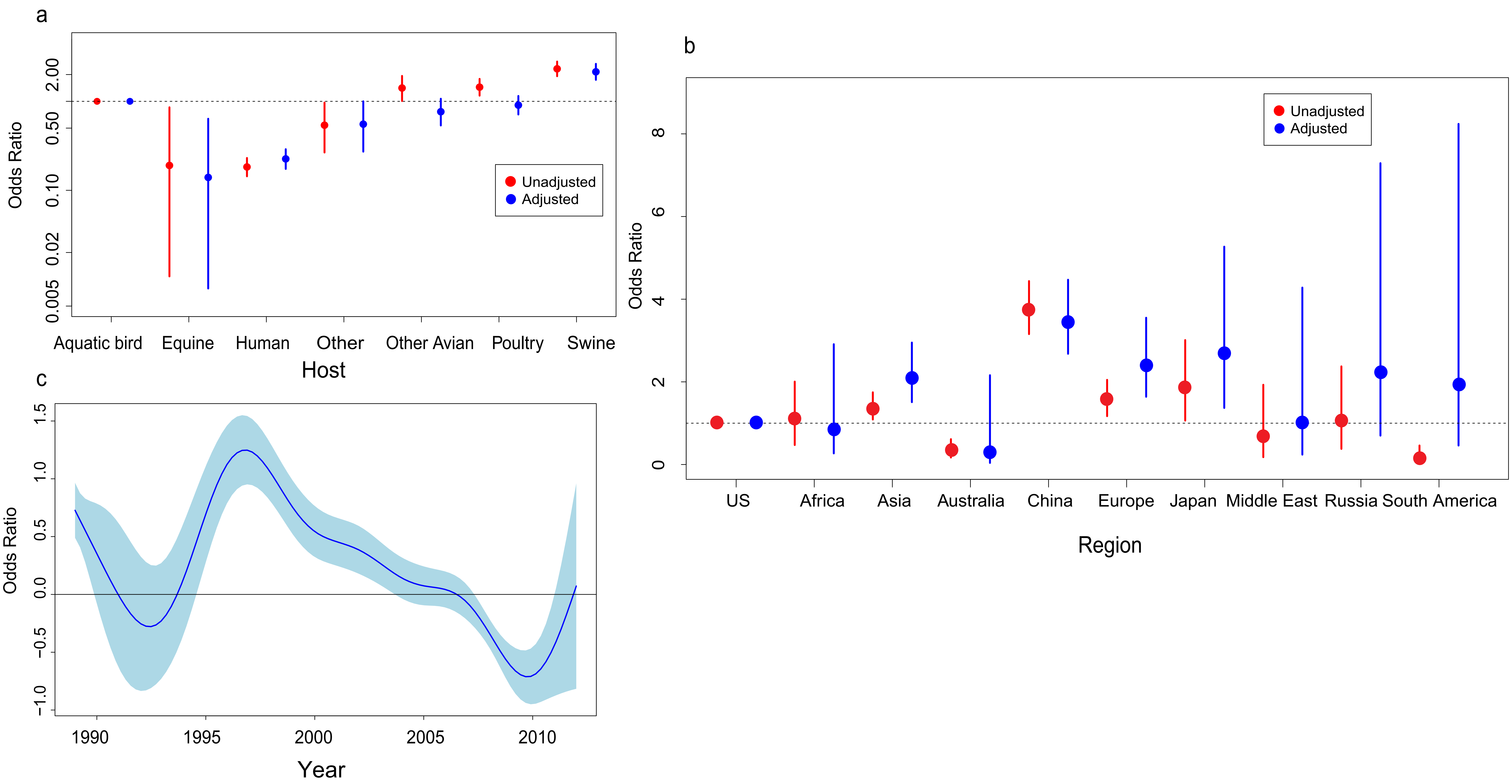

Supplement: Supplementary file 6 — Sensitivity analysis to regression results. (PDF 422 kb) [file 12879_2015_1298_MOESM6_ESM.pdf]
