# Supplementary figures and images for: A systematic review of reported reassortant viral lineages of influenza A
Source: BMC Infect Dis. 2016 Jan 5;16:3. doi: 10.1186/s12879-015-1298-9 (PMC4702296; doi:10.1186/s12879-015-1298-9)

# H1 Swine

- Reassorted data
- Random sample data

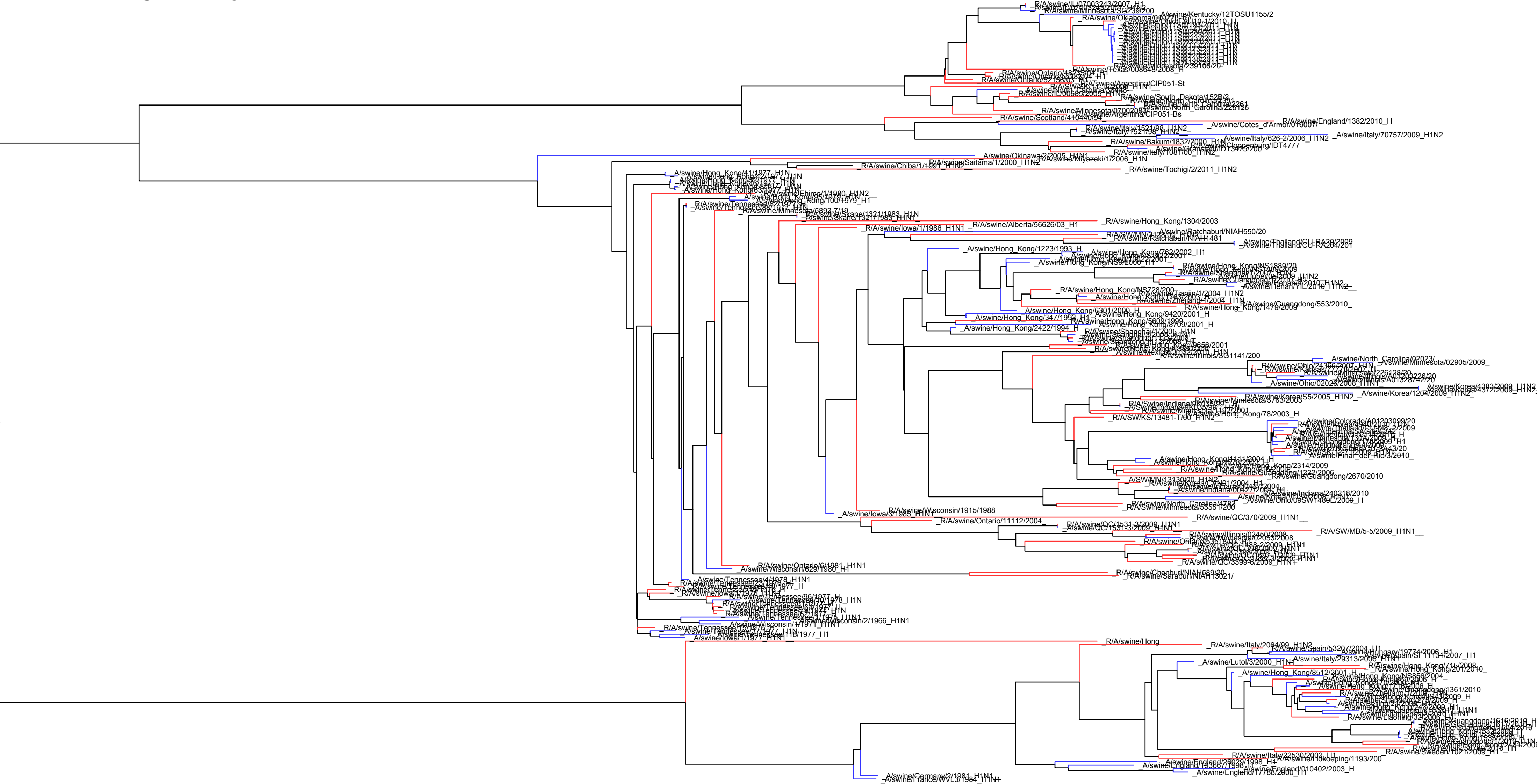

# H5 Avian

Reassorted data

— Random sample data

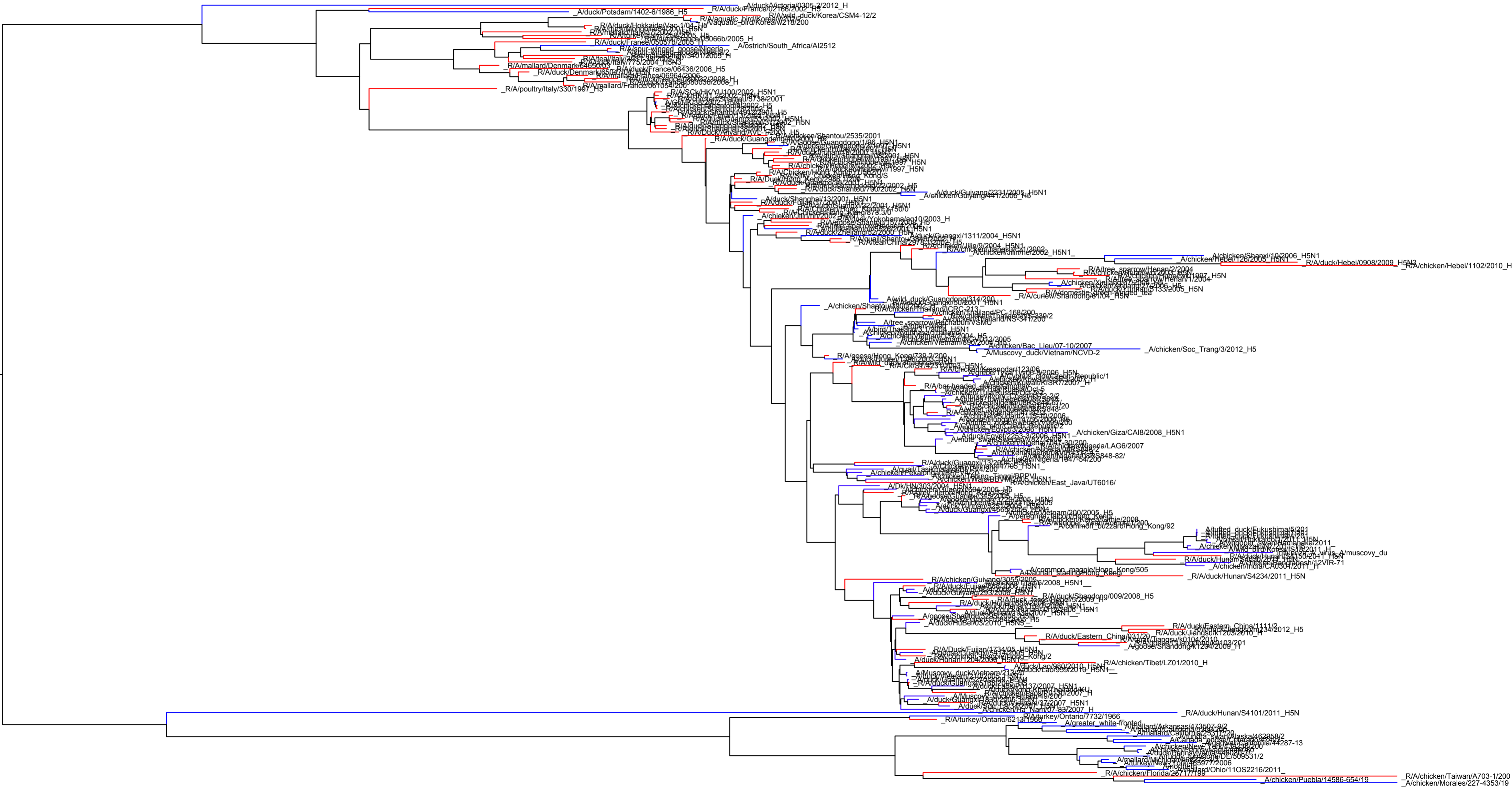

0.05

Supplement: Supplementary file 7 — Labelled phylogenies. (PDF 414 kb) [file 12879_2015_1298_MOESM7_ESM.pdf]
